# MHC-Related Shared Genetic Susceptibility Between Ulcerative Colitis and Gastric Cancer in East Asians

**DOI:** 10.3390/genes17091077

**Published:** 2026-09-07

**Authors:** Ji Eun Kim, Hyuk Lee, Young Eun Oh, Tae-Se Kim, Yang Won Min, Byung-Hoon Min, Jun Haeng Lee, Poong-Lyul Rhee

**Affiliations:** 1 Department of Medicine, Samsung Medical Center, Sungkyunkwan University School of Medicine, 81 Irwon-Ro, Gangnam-Gu, Seoul 06351, Republic of Korea; 2 Samsung Precision Genome Medicine Institute, Samsung Medical Center, 81 Irwon-Ro, Gangnam-Gu, Seoul 06351, Republic of Korea

**Keywords:** gastric cancer, genetic epidemiology, *HLA-DQA1/DQB1*, major histocompatibility complex, Mendelian randomization, ulcerative colitis

## Abstract

Background/Objectives: Observational studies in East Asia have not shown increased gastric cancer risk in ulcerative colitis, whereas a previous genetic analysis reported a positive association. Because both diseases have major histocompatibility complex (MHC) signals, we assessed whether the association was most apparent in MHC variants under ancestry-matched instrument selection. Methods: We performed a two-stage, two-sample Mendelian randomization study of 12 East Asian datasets (11 immune-related phenotypes) and a gastric cancer genome-wide association study (7921 cases; 159,201 controls), with instruments selected against the 1000 Genomes East Asian panel. For ulcerative colitis, we compared all, MHC, and non-MHC variants, evaluated signal overlap at *HLA-DQA1/DQB1*, and assessed cross-disease specificity. Results: Ulcerative colitis was the only phenotype associated with gastric cancer after false-discovery-rate correction (odds ratio [OR] 1.16, 95% confidence interval [CI] 1.09–1.24; q = 1.37 × 10^−4^). The MHC estimate was similar (OR 1.18, 95% CI 1.11–1.26), whereas the non-MHC estimate was close to 1 but rested on only two variants and remained imprecise (OR 1.00, 95% CI 0.86–1.17). The traits showed overlapping signals at *HLA-DQA1/DQB1*, and the MHC variants were associated with several other immune diseases. Conclusions: The positive association was most apparent in MHC variants that were not specific to ulcerative colitis. The association therefore appears to be largely carried by pleiotropic MHC variants, although these data cannot establish the pathway that explains it.

## 1. Introduction

Gastric cancer remains a major clinical burden in East Asia, and its development reflects interactions between environmental exposures and host immune responses [1,2,3]. These overlapping environmental and immune influences complicate genetic interpretation because the same variants may affect several related pathways.

Observational studies have linked immune-mediated diseases to gastric cancer, but the direction and magnitude of these associations vary across diseases and populations [4,5,6]. In a nationwide Korean cohort, patients with newly diagnosed ulcerative colitis had a lower risk of gastric cancer than matched comparators (adjusted hazard ratio 0.60, 95% CI 0.47–0.77) [7]. A previous Mendelian randomization study based on European-ancestry data found no evidence of causal associations between genetic susceptibility to any of 12 autoimmune diseases and gastric cancer risk and suggested that shared genetic architecture or other unmeasured pathways might explain the epidemiologic associations [8]. By contrast, an East Asian Mendelian randomization analysis that used the same ulcerative colitis dataset and the Biobank Japan gastric cancer dataset included in our sensitivity analysis reported a positive association (OR 1.124, 95% CI 1.062–1.189) but did not determine whether MHC variants accounted for the estimate [9]. Together, these contrasting epidemiologic and genetic findings leave it unclear whether the East Asian association reflects ulcerative colitis itself or variation that influences both diseases through separate pathways.

Ulcerative colitis provides a useful setting in which to examine this question because it has strong genetic associations in the HLA region [10], yet no established mechanism directly links the disease itself to gastric cancer. Mendelian randomization can support causal inference only when the selected variants are associated with the exposure, are independent of relevant confounders, and affect the outcome solely through the exposure [11]. These requirements are difficult to satisfy in the extended MHC, where variants are densely correlated and associated with numerous immune and infectious phenotypes [12]. Host genetic associations with *Helicobacter pylori* susceptibility have also been reported at HLA-related loci [13]. Thus, an MHC variant used as an instrument for ulcerative colitis could be associated with gastric cancer through an infection-related or other immune pathway even if ulcerative colitis itself is not causal.

We therefore reexamined the reported genetic association between ulcerative colitis and gastric cancer in East Asian populations. We first screened immune-related phenotypes to place the finding in a broader context. We then examined whether the estimate persisted outside the MHC, whether ulcerative colitis and gastric cancer had overlapping regional signals at *HLA-DQA1/DQB1*, and whether the selected variants were also associated with related intestinal diseases, other immune diseases, and non-immune traits. These analyses were designed to distinguish a disease-specific relationship from shared MHC-related susceptibility without assuming that ulcerative colitis causes gastric cancer.

## 2. Materials and Methods

### 2.1. Study Design and Reporting

**Figure 1:**
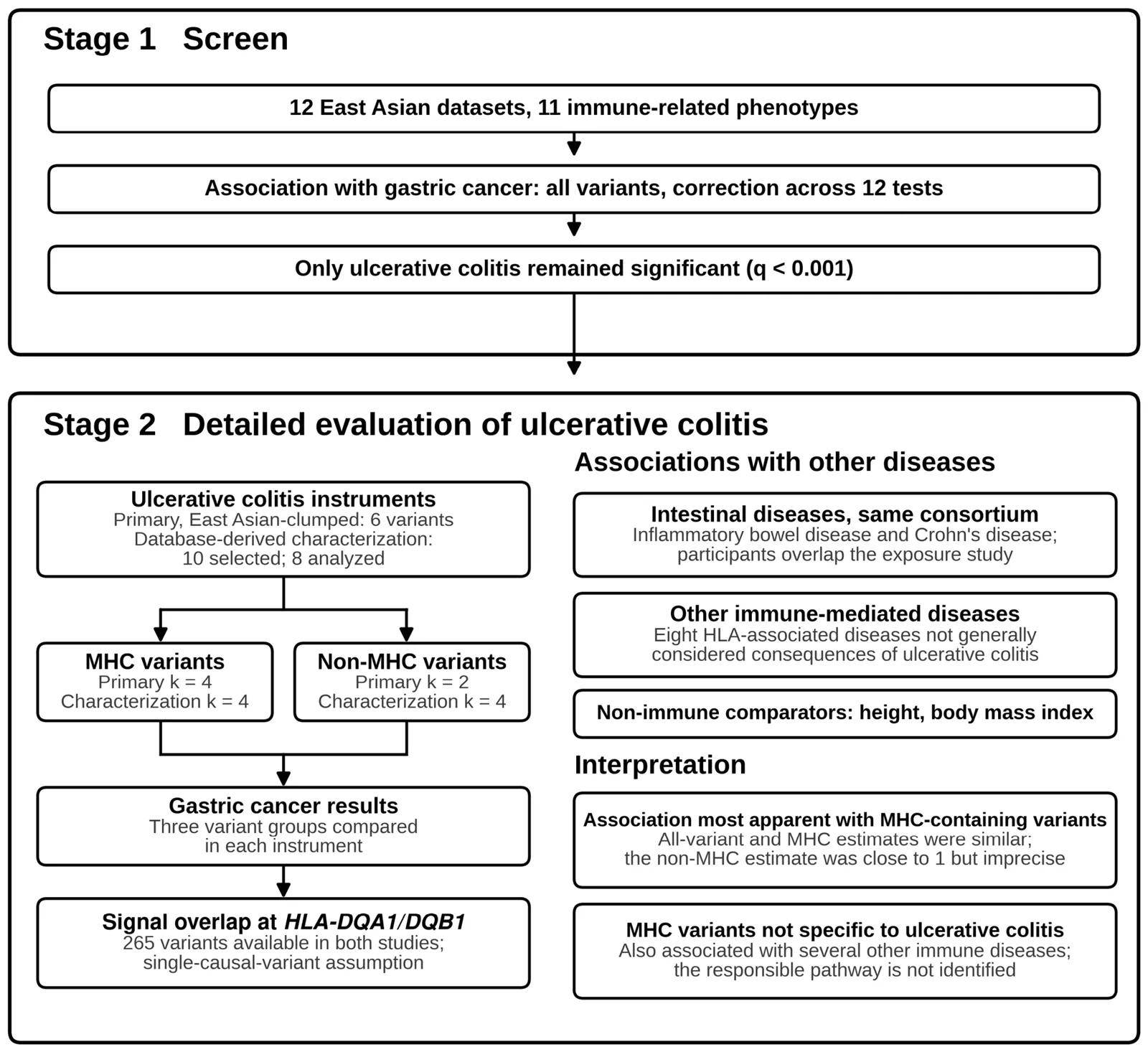
Two-stage study design and interpretation. Stage 1 screened 12 East Asian datasets using instruments selected against an explicit East Asian linkage disequilibrium reference panel. Ulcerative colitis was the only association that remained significant after correction. Stage 2 compared the primary East Asian-clumped instrument with a database-derived characterization instrument, partitioned variants by MHC status, assessed regional overlap at *HLA-DQA1/DQB1*, and examined cross-disease specificity. The results indicate that the association is largely carried by pleiotropic MHC variants rather than demonstrating an ulcerative-colitis-mediated effect.

We conducted a two-stage study using publicly available genetic association summary statistics and followed STROBE-MR reporting guidance for the Mendelian randomization components (checklist in Table S1, Supplementary Materials) [11]. Stage 1 screened 12 datasets representing 11 immune-related phenotypes using instruments selected against the 1000 Genomes East Asian linkage disequilibrium reference panel. Stage 2 evaluated ulcerative colitis in detail because it was the only association that remained significant after correction. The East Asian-clumped instrument was primary for the screen and the principal gastric cancer comparison. The original database-derived instrument was retained for expanded variant-level characterization because it included more non-MHC variants and was also evaluated as a sensitivity instrument. Ulcerative colitis was selected after the screen rather than specified in advance. The study protocol was not preregistered, and the complete screening results are reported in the Supplementary Materials. Data were accessed and verified in July 2026. All analyses used deidentified summary statistics, and no additional ethical approval was required. Figure 1 summarizes the study design and interpretation.

### 2.2. Data Sources and Focused Analysis Set

The primary gastric cancer dataset comprised 7921 cases and 159,201 controls of East Asian ancestry from Biobank Japan (GWAS Catalog GCST90018629) [14]. A second Biobank Japan dataset (6563 cases and 195,745 controls; bbj-a-119) was used only to assess consistency when data were available. It was not considered an independent replication because its participants overlapped with those in the primary dataset [15]. The ulcerative colitis dataset comprised 1134 cases and 3719 controls of East Asian ancestry from the International Inflammatory Bowel Disease Genetics Consortium (ieu-a-969) [10]. Table 1 summarizes the principal datasets; the complete screening panel and results are provided in Tables S2–S5 and Figure S1, Supplementary Materials. Because the ulcerative colitis and primary gastric cancer datasets were generated by different consortia, participant overlap was unlikely. The ulcerative colitis study used the Immunochip, which covers approximately 186 established immune-related regions rather than the whole genome. This design limited the variants available for selection, which we considered when interpreting the results.

### 2.3. Variant Selection and MHC Partitioning

For the database-derived characterization instrument, variants associated with each exposure at *p* < 5 × 10^−8^ were selected from OpenGWAS and clumped at r^2^ < 0.001 within 10,000 kb. The reference population used by the database interface for the initial clumping call was not recorded, and the East Asian option in that interface returned the same variants as the European option. We therefore repeated variant selection using an explicit East Asian reference panel, first for the ulcerative colitis instrument and then for all 12 datasets in the initial screen so that the screen itself could be repeated on an ancestry-matched basis. All variants associated with ulcerative colitis at *p* < 5 × 10^−8^ were retrieved without clumping and were clumped again at r^2^ < 0.001 within 10,000 kb using the 1000 Genomes East Asian panel [16], with variants matched by their GRCh37 coordinates. We refer to the explicitly East Asian-clumped set as the primary instrument and the original database-derived set as the characterization instrument. The primary instrument was used for the complete 12-dataset screen and the principal gastric cancer comparison. The characterization instrument, which retained more non-MHC variants, was used for expanded MHC and non-MHC analyses and as a sensitivity check. The primary ulcerative colitis instrument contained six variants (four MHC and two non-MHC), whereas the characterization instrument contained eight analyzed variants (four MHC and four non-MHC). We also quantified the pairwise correlations among the selected variants in the same panel. Variants with ambiguous strand alignment and variants unavailable in the gastric cancer dataset were removed during harmonization. The extended MHC was defined as chromosome 6:25–35 Mb in GRCh37. We evaluated three groups: all variants, MHC variants, and variants outside the MHC. Ten ulcerative colitis variants were initially selected for the database-derived characterization instrument. rs2940716 was unavailable in the gastric cancer data, and rs13203429 had ambiguous allele alignment, leaving four MHC and four non-MHC variants. For the primary outcome, rs13397737 was represented by proxy rs55770424. The two variants are 23,098 bp apart and were strongly correlated in the 1000 Genomes European reference panel (r^2^ = 0.966), but the queried East Asian panels did not yield an LD estimate. We therefore repeated the analysis after excluding this proxy. Variant strength was calculated as F = (betaX/SEbetaX)^2^. For the database-derived characterization instrument, minimum F statistics were 31.8 for all variants, 31.8 for MHC variants, and 32.7 for variants outside the MHC; for the East Asian-clumped primary instrument, the corresponding minima were 32.7, 50.2, and 32.7. Variant-level details, including the exposure effect estimates and F statistics for both instruments, are provided in Tables S6 and S23, Supplementary Materials. Because the exposure study used a targeted immune array, the non-MHC group does not represent a complete genome-wide set. The primary non-MHC analysis therefore contained only two variants, and the expanded characterization contained four. Because these variants were derived from an Immunochip dataset rather than from genome-wide genotyping, non-MHC coverage was incomplete and may not represent the full spectrum of ulcerative colitis susceptibility; this substantially limits the central comparison between MHC and non-MHC instruments.

We assessed the three core requirements of Mendelian randomization. Relevance was assessed using F statistics. Independence from confounders could not be established from summary data. We assessed ancestry alignment but used cross-trait associations to examine instrument specificity, not to rule out confounding. The requirement that variants affect gastric cancer only through ulcerative colitis was especially uncertain within the MHC. We therefore evaluated MHC and non-MHC variants separately and examined variation among individual estimates, evidence of alternative pathways, and regional signal overlap. Attenuation of the point estimate after removing MHC variants was interpreted descriptively as evidence that the MHC contributed to the overall result, not as proof of a direct effect or a formal difference between variant groups. In the 1000 Genomes East Asian panel, the largest pairwise r^2^ among the four MHC variants of the database-derived characterization instrument was 0.019. Sensitivity analyses used the corresponding signed correlations after alignment to the exposure effect alleles. For the eight-variant model, the measured 4 × 4 MHC block was incorporated into an identity correlation matrix; correlations involving non-MHC variants were therefore assumed to be zero. Accounting for the measured MHC correlations had little effect on the odds ratios (Tables S7 and S8, Supplementary Materials).

### 2.4. Association Estimation and Sensitivity Analyses

Multiplicative random-effects inverse-variance-weighted (IVW) regression was the primary estimator; the residual standard error was constrained to at least 1, so analyses with underdispersion reduced to fixed-effect IVW. Odds ratios represent the change in gastric cancer odds per one-unit increase in the log odds of genetic susceptibility to ulcerative colitis; these estimates do not represent the effect of being diagnosed with ulcerative colitis [17]. Weighted-median [18] and MR–Egger slope [19] estimates were calculated as alternative estimators. Cochran’s Q assessed heterogeneity, and the MR–Egger intercept [19], MR-PRESSO global test [20], and Steiger directionality test [21] were reported when estimable. MR-PRESSO used 100,000 simulations, and the global, outlier, and distortion tests were reported. Correlation-aware IVW analyses used generalized models in MendelianRandomization with the signed matrix described above. Because the selected variants may influence several immune pathways, a statistically significant estimate was not interpreted as evidence of causality. Both the all-variant and non-MHC analyses were repeated after excluding the proxy. Full results are reported in Table S9, Supplementary Materials. Leave-one-out analysis and single-variant Wald ratio estimates were computed for each variant group to assess whether one variant accounted for the result. Using the observed standard error, we calculated the minimum detectable odds ratio with 80 percent power. We also attempted the reverse direction, using gastric cancer liability as the exposure and ulcerative colitis as the outcome.

The Benjamini–Hochberg false discovery rate correction was applied to the 12 all-variant tests in the primary East Asian-clumped screen and, separately, to the repeated database-derived screen. MHC-only and non-MHC analyses were secondary. Exploratory comparisons used a zero-covariance approximation, a nonparametric bootstrap, and a label-permutation test. These comparisons were performed in the database-derived characterization analysis, in which each group contained four variants; because both groups used the same gastric cancer dataset, the resulting *p* values varied substantially across methods. No formal comparison was attempted for the primary East Asian-clumped instrument, which contained only two non-MHC variants. We therefore interpreted the group-specific estimates and confidence intervals descriptively and did not infer a formal difference between groups. No a priori power calculation was performed because all qualifying variants in the available datasets were used; precision was assessed using the 95% confidence intervals, and the non-MHC estimate was not interpreted as evidence that an effect was absent. Given the small number of non-MHC variants and the incomplete non-MHC coverage of the exposure array, any divergence between the MHC and non-MHC groups was described as a pattern rather than as a statistically demonstrated difference.

### 2.5. Regional Overlap of Association Signals

Association patterns for ulcerative colitis and gastric cancer were evaluated using coloc.abf [22] across chromosome 6:32,450,000–32,800,000 (GRCh37), which spans *HLA-DQA1/DQB1*. The analysis included all 265 variants available for both diseases in the region, without selection on statistical significance, and used the default coloc.abf prior probabilities (p1 = p2 = 1 × 10^−4^ and p12 = 1 × 10^−5^). The sensitivity analyses varied p12 while holding p1 and p2 fixed at 1 × 10^−4^, and varied p1 and p2 jointly while holding p12 fixed at 1 × 10^−5^. The method estimates posterior probabilities for five hypotheses, including distinct signals and a shared signal under a single-causal-variant model. All five probabilities are reported. Because the MHC commonly contains multiple correlated signals, a high probability of a shared signal was considered evidence of regional overlap only, not proof of a single causal allele or a pathway through ulcerative colitis. The analysis was repeated across a range of prior probabilities and, separately, in windows of 100 kb to 1 Mb on each side of the lead ulcerative colitis variant, to assess the result across different prior specifications and analysis windows (Tables S10–S12, Supplementary Materials). Regional coverage differed markedly: 280 variants were available for ulcerative colitis and 13,093 for gastric cancer because the ulcerative colitis study used a targeted array. The ulcerative colitis data were therefore too sparse to resolve multiple independent signals reliably. The single-causal-variant assumption underlying coloc.abf is difficult to justify in the MHC, where multiple correlated signals are common. Together with the sparse regional coverage of the exposure data and the sensitivity of the posterior probability to the prior specification, this means that the analysis can support regional overlap but cannot provide strong evidence for a single shared causal signal.

### 2.6. Analysis of Associations with Intestinal and Other Immune Diseases

We examined associations of the MHC and non-MHC variant groups with additional outcomes. East Asian inflammatory bowel disease and Crohn’s disease were included as within-consortium checks, not independent confirmation, because their datasets included overlapping cases or shared controls with the ulcerative colitis study. A European ulcerative colitis dataset was used to examine transferability across ancestries. Eight other HLA-associated immune diseases were analyzed to assess whether the selected variants were specific to ulcerative colitis; associations with diseases that are not expected to be consequences of ulcerative colitis would indicate broader immune effects. Height and body mass index were included as non-immune comparisons. Benjamini–Hochberg correction was applied to the 16 MHC and non-MHC comparisons across the eight immune diseases.

Outcome datasets were identified by searching trait names in OpenGWAS, prioritizing East Asian datasets with the largest available case counts. Table S13, Supplementary Materials, lists the selected datasets, ancestries, and sample sizes. Most East Asian outcomes came from the same Biobank Japan release and were therefore correlated. The intestinal disease datasets also shared participants with the ulcerative colitis study. These analyses were used to describe the cross-disease behavior of the selected variants, not as independent confirmation of the gastric cancer association.

### 2.7. Software and Reproducibility

Analyses were performed in R version 4.6.1 (R Foundation for Statistical Computing, Vienna, Austria) using TwoSampleMR (version 0.7.9), MendelianRandomization (https://cran.r-project.org/package=MendelianRandomization, accessed on 31 July 2026), ieugwasr (version 1.1.0), MR-PRESSO (https://github.com/rondolab/MR-PRESSO, accessed on 31 July 2026), and coloc (https://cran.r-project.org/package=coloc, accessed on 31 July 2026); OpenGWAS data access followed the MR-Base platform framework [23]. The archived analysis repository provides the analysis scripts, the derived variant-level inputs, all output tables, the random seeds, and a table mapping each analytical output to the corresponding result in this article and in Supplementary Materials (https://doi.org/10.5281/zenodo.21736825). Full genome-wide summary statistics are not redistributed because they remain subject to source-database licenses. Each of the ten variants selected for the database-derived characterization instrument was checked against the GRCh37 file distributed by the GWAS Catalog for GCST90018629. Chromosome, position, alleles, allele frequency, beta, and standard error matched exactly for the eight variants present in that file; rs13397737 and rs2940716 were absent from it. The proxy record used in place of rs13397737 was supplied by the outcome database and was not independently verified against the distributed file; analyses excluding this proxy are reported separately and are not a substitute for that verification. Harmonised outcome records for the variants of the East Asian-clumped primary instrument are provided in the archived repository as R1_harmonised_new_instrument.csv. An alternative identifier-based lookup returned neighboring variants in two instances; this was treated as a database-retrieval sensitivity check, and for those two variants, rs2844623 and rs10975003, the records retained for analysis were confirmed against the distributed file (Tables S14 and S15, Supplementary Materials).

## 3. Results

### 3.1. Initial Immune-Phenotype Screen

Using the primary East Asian-clumped instruments, ulcerative colitis was the only phenotype associated with gastric cancer after correction across the 12 datasets (OR 1.163, 95% CI 1.087–1.244; *p* = 1.1 × 10^−5^; q = 1.37 × 10^−4^). The next smallest corrected value was 0.347, and no exposure changed its significance verdict relative to the database-derived screen. In that repeated screen, ulcerative colitis was again the only association that remained significant (OR 1.134, 95% CI 1.068–1.205; q = 5.27 × 10^−4^). The complete results from both instrument-selection approaches are reported in Tables S3–S5 and Figure S1, Supplementary Materials. Subsequent analyses therefore focused on ulcerative colitis.

### 3.2. Gastric Cancer Results After Separating MHC and Non-MHC Variants

**Figure 2:**
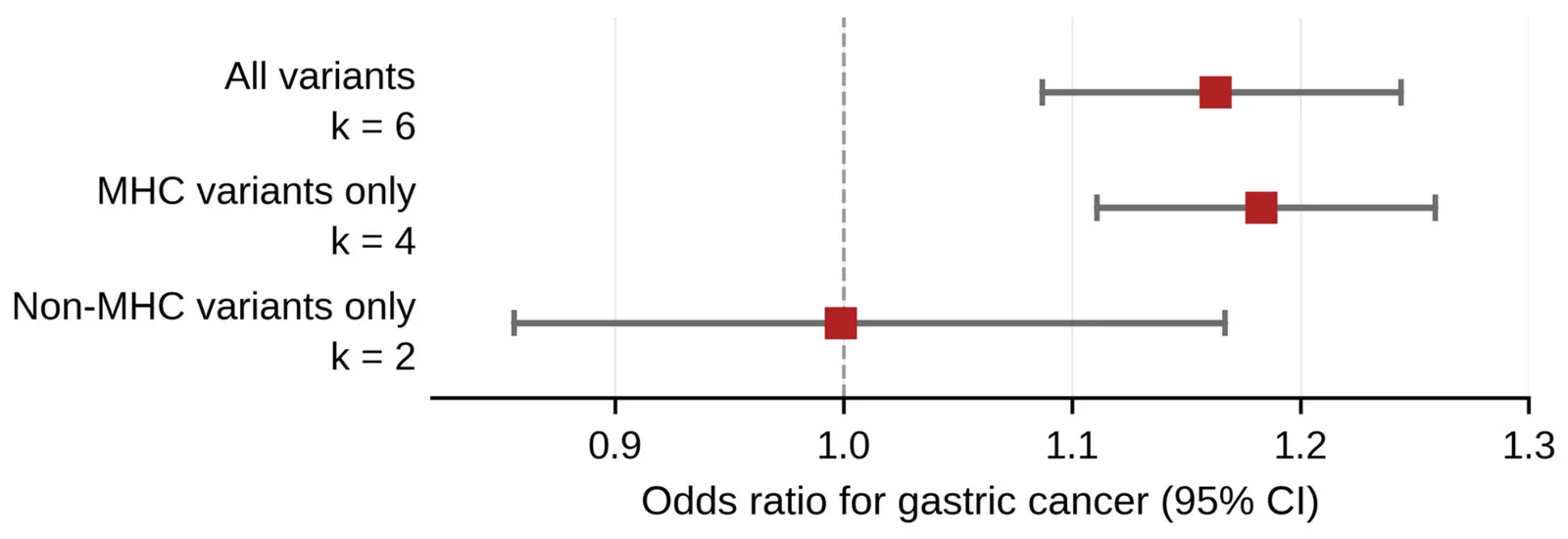
Primary gastric cancer estimates for genetic susceptibility to ulcerative colitis using the East Asian-clumped instrument. Squares indicate odds ratios, horizontal lines indicate 95% confidence intervals, and k is the number of variants analyzed. The MHC estimate was similar to the all-variant estimate. The two-variant non-MHC estimate was close to 1 but imprecise. Results for the database-derived characterization instrument, which contained four MHC and four non-MHC variants, are reported in Table 2 and in Table S17, Supplementary Materials.

With the primary East Asian-clumped ulcerative colitis instrument, greater genetic susceptibility to ulcerative colitis was associated with higher gastric cancer odds (k = 6; OR 1.163, 95% CI 1.087–1.244; *p* = 1.1 × 10^−5^). The four MHC variants produced a similar estimate (OR 1.183, 95% CI 1.111–1.259; *p* = 1.4 × 10^−7^), whereas the estimate from the two variants outside the MHC was close to 1 and imprecise (OR 0.999, 95% CI 0.856–1.167; *p* = 0.99). These primary estimates are presented in Figure 2, Table 2 and Table S16, Supplementary Materials.

In the expanded characterization using the database-derived instrument, the four MHC variants were associated with higher gastric cancer odds, with an estimate similar to the all-variant result (OR 1.154, 95% CI 1.085–1.227; *p* = 4.92 × 10^−6^). Accounting for correlations among these variants produced an almost identical estimate (OR 1.154, 95% CI 1.077–1.235; *p* = 4.25 × 10^−5^). The estimate based on four variants outside the MHC was close to 1 (OR 0.984, 95% CI 0.857–1.130; *p* = 0.823). The non-MHC weighted-median estimate was also close to 1 (OR 1.02; *p* = 0.80), and the MR–Egger slope was imprecise (OR 0.68; *p* = 0.25). Excluding the proxy from the all-variant analysis produced a similar estimate (k = 7; OR 1.136, 95% CI 1.072–1.204; *p* = 1.48 × 10^−5^). Excluding it from the non-MHC analysis also had little effect (k = 3; OR 0.998, 95% CI 0.887–1.122; *p* = 0.972). The confidence intervals from both non-MHC IVW analyses were compatible with modestly lower or higher gastric cancer odds. Exploratory comparisons of the MHC and non-MHC estimates were unstable and method-dependent and did not establish a formal difference between the groups; because only two and four non-MHC variants were available, this contrast is reported as a pattern rather than as a statistically demonstrated difference (Table S4 and its footnote, Supplementary Materials). Full results with 95% confidence intervals for this instrument are reported in Table S17, Supplementary Materials.

Odds ratios are dispersion-constrained inverse-variance-weighted estimates. The East Asian-clumped instrument is primary. The database-derived instrument is retained for expanded characterization and sensitivity analysis. MHC and non-MHC estimates are interpreted descriptively because formal comparisons were method-dependent.

### 3.3. Overlap of Association Signals in HLA-DQA1/DQB1

**Figure 3:**
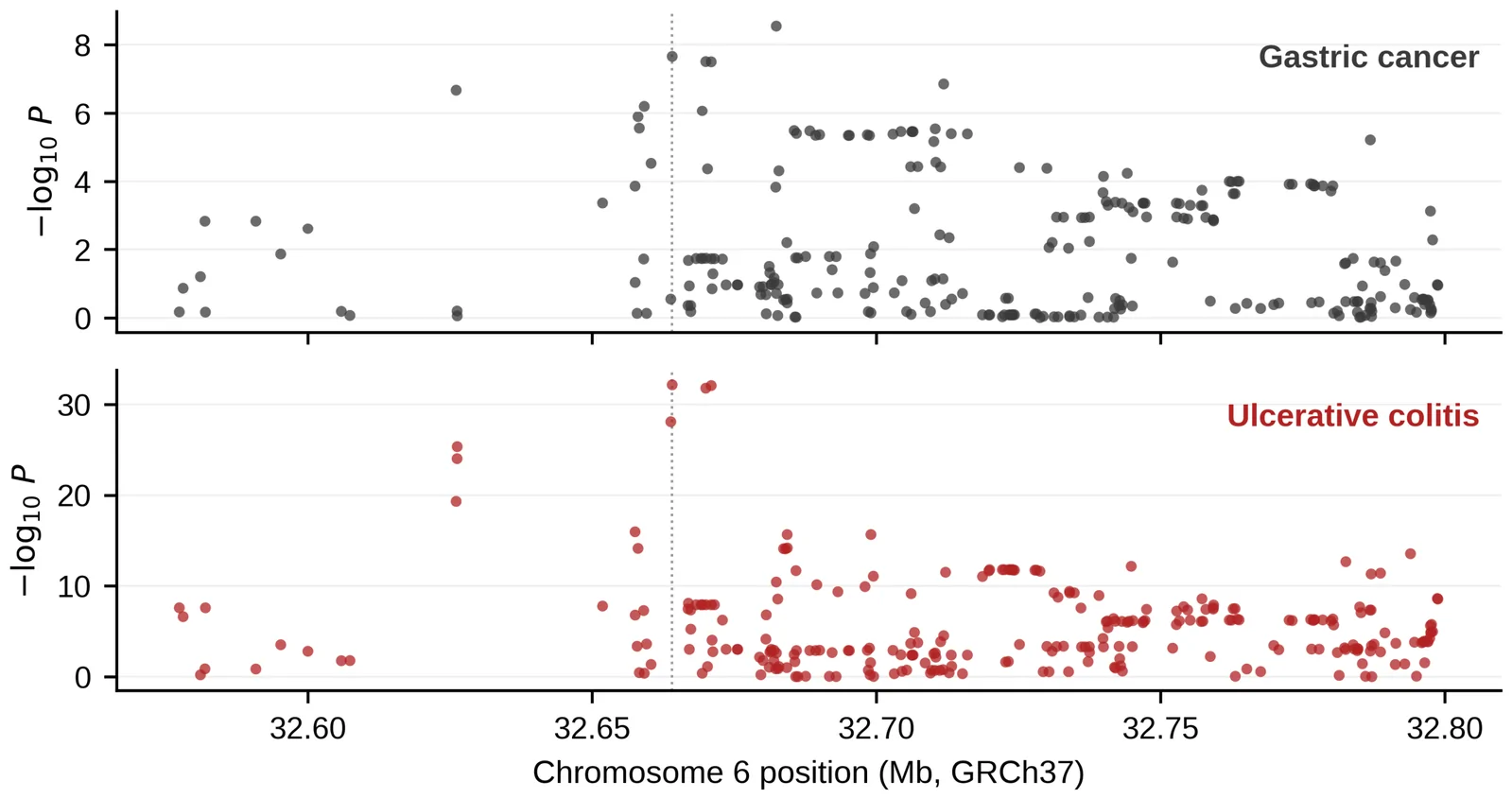
Ulcerative colitis and gastric cancer association signals at *HLA-DQA1/DQB1*. Both panels show the same 265 variants available in both studies within the chromosome 6 region (GRCh37); the vertical-axis scales differ. The vertical dotted line in each panel marks the position of the lead ulcerative colitis variant, rs2647015. Under the single-causal-variant model, the posterior probability of a shared regional signal was 0.989, and the probability of distinct signals was 0.011. These findings support regional overlap but do not identify a single causal variant or demonstrate that ulcerative colitis mediates the shared signal; the posterior probability was also sensitive to the prior specification and to the single-causal-variant assumption, which is difficult to justify in the MHC. Full posterior probabilities and prior sensitivity analyses are provided in Tables S10–S12, Supplementary Materials.

In the coloc.abf analysis, the posterior probability of a shared regional signal under the single-causal-variant assumption was 0.989, and the probability of distinct signals was 0.011. The ulcerative colitis and gastric cancer signals overlapped in the analyzed *HLA-DQA1/DQB1* region. Support for overlap remained high across the tested range of p12 but fell to 0.48 when the single-trait priors p1 and p2 were increased to 1 × 10^−3^, indicating that the posterior probability was sensitive to prior assumptions. The analysis cannot identify a single causal allele or establish ulcerative colitis as the pathway to gastric cancer. Widening the analysis window did not change this conclusion: across windows from 100 kb to 1 Mb on each side of rs2647015, which was the lead variant in every window, the posterior probability of a shared signal ranged from 0.90 to 0.99 and declined as the window widened. The result nevertheless depends on variant coverage, linkage disequilibrium, prior probabilities, and the single-causal-variant assumption, which is difficult to justify in the MHC. These findings therefore support regional overlap between the two traits but do not provide strong evidence for a single shared causal signal. Complete results are provided in Figure 3 and Tables S10–S12, Supplementary Materials.

### 3.4. Associations with Intestinal and Other Immune Diseases

**Figure 4:**
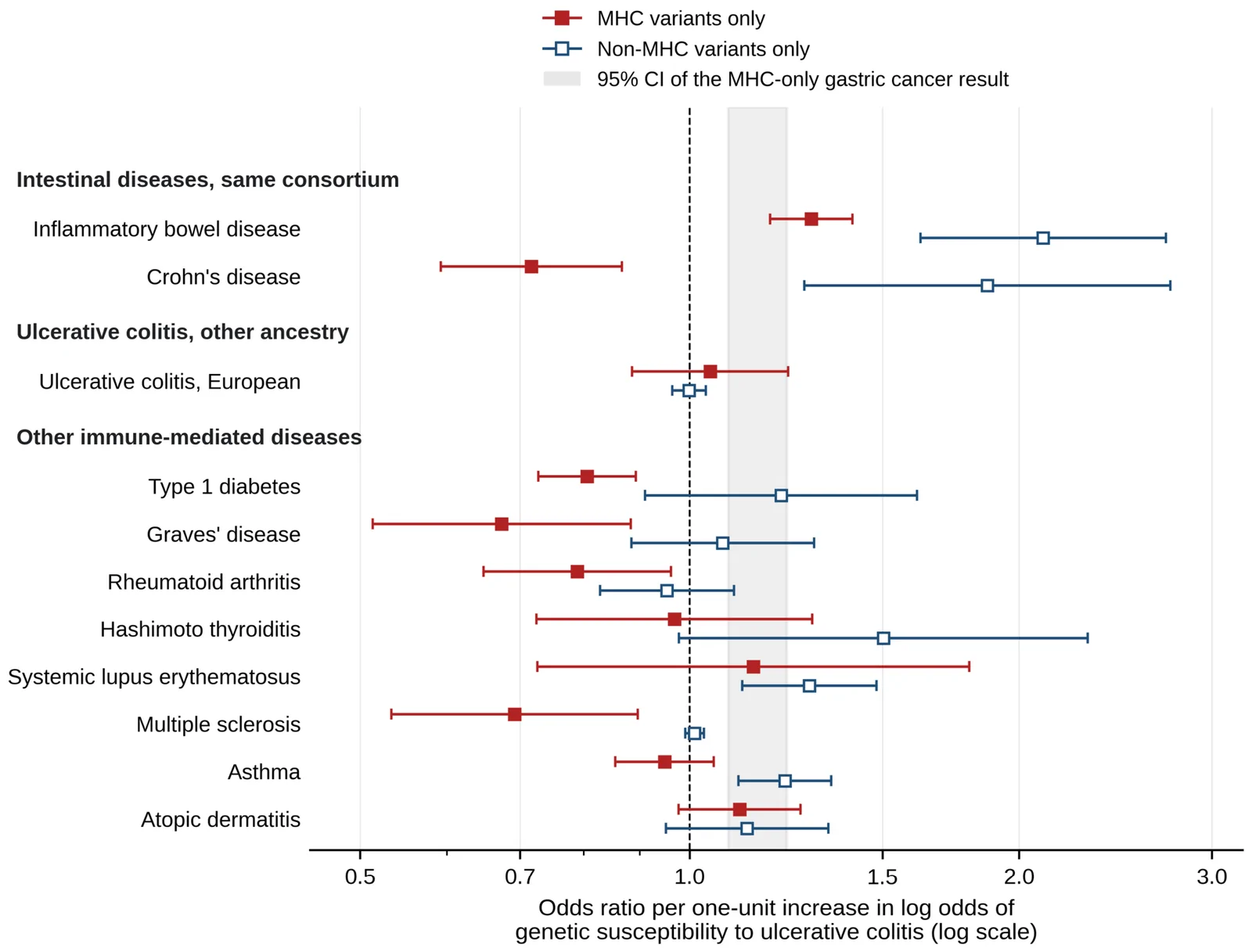
Expanded characterization of associations between database-derived MHC and non-MHC ulcerative-colitis-associated variant groups and other outcomes. Squares indicate odds ratios, and horizontal lines indicate 95% confidence intervals. The inflammatory bowel disease and Crohn’s disease datasets overlap with the ulcerative colitis study and serve only as within-consortium checks. The European ulcerative colitis dataset assesses cross-ancestry transferability. Multiple-testing-adjusted q values are reported in Table S18, Supplementary Materials. Height and body mass index are reported in Table S18.

In East Asian datasets from the same consortium, variants outside the MHC were associated with inflammatory bowel disease (OR 2.10, 95% CI 1.63–2.72; *p* = 1.7 × 10^−8^) and Crohn’s disease (OR 1.87, 95% CI 1.27–2.75; *p* = 1.4 × 10^−3^). MHC variants were associated with inflammatory bowel disease (OR 1.29, 95% CI 1.19–1.41; *p* = 6.5 × 10^−9^) and lower odds of Crohn’s disease (OR 0.72, 95% CI 0.59–0.87; *p* = 6.2 × 10^−4^). Because these datasets shared cases or controls with the ulcerative colitis study, the findings were treated as within-consortium consistency checks rather than independent confirmation. Neither variant group showed a clear association with ulcerative colitis in the European dataset. Because variant effects and linkage disequilibrium can differ by ancestry, this cross-ancestry difference was interpreted as evidence of limited transferability rather than as a failed independent replication (Figure 4 and Table S18, Supplementary Materials).

After correction for multiple testing, MHC variants were associated with type 1 diabetes (OR 0.81; q = 4.67 × 10^−4^), Graves’ disease (OR 0.67; q = 0.017), rheumatoid arthritis (OR 0.79; q = 0.050), and multiple sclerosis (OR 0.69; q = 0.017). Variants outside the MHC were associated with systemic lupus erythematosus (OR 1.29; q = 0.0025) and asthma (OR 1.22; q = 4.67 × 10^−4^). These findings indicate that the selected ulcerative-colitis-associated variants were not specific to ulcerative colitis; they do not imply that ulcerative colitis affects the risk of those diseases.

The directions of association differed across immune diseases. These cross-disease associations indicate limited specificity for ulcerative colitis but do not distinguish mediation through ulcerative colitis from other pathways. Neither variant group was associated with height or body mass index (all *p* ≥ 0.58). These comparisons do not identify the pathway that links the variants to gastric cancer (Table S18, Supplementary Materials).

### 3.5. Instrument Selection and Sensitivity Analyses

With the database-derived characterization instrument, leave-one-out analyses showed that the odds ratio ranged from 1.099 to 1.151 and every estimate remained statistically significant; omitting rs2647015, the variant with the strongest individual association, produced the lowest value (OR 1.099, 95% CI 1.030–1.173; *p* = 0.004). Individually, three MHC variants were nominally associated with gastric cancer (rs2647015, OR 1.215, *p* = 2.2 × 10^−8^; rs2844623, OR 1.186, *p* = 1.7 × 10^−5^; rs7741807, OR 1.087, *p* = 0.032), whereas no variant outside the MHC was. These single-variant *p* values are unadjusted. For the non-MHC group, no leave-one-out estimate was statistically significant. For this characterization instrument, the observed standard errors corresponded to minimum detectable odds ratios of about 1.09 for the all-variant and MHC-only groups and 1.22, or equivalently 0.82, for the non-MHC group. For the primary East Asian-clumped instrument, the corresponding value for the two-variant non-MHC group was about 1.25, or equivalently 0.80. Smaller non-MHC effects could therefore not be excluded in either instrument. Complete leave-one-out and single-variant results are provided in Tables S19–S21 and Figure S2, Supplementary Materials.

Selection with the explicit East Asian reference panel, used for the primary analysis, substantially changed the instrument composition relative to the database-derived characterization instrument. Of 561 variants associated with ulcerative colitis at *p* < 5 × 10^−8^, clumping at r^2^ < 0.001 within 10,000 kb retained six variants, four in the MHC and two outside it, and only three of these were in the original instrument. The gastric cancer estimates were nevertheless similar: OR 1.163 (95% CI 1.087–1.244; *p* = 1.1 × 10^−5^) for all variants, OR 1.183 (95% CI 1.111–1.259; *p* = 1.4 × 10^−7^) for MHC variants, and OR 0.999 (95% CI 0.856–1.167; *p* = 0.99) for the two variants outside the MHC. Point estimates differed by no more than 2.6 percent, were directionally consistent, and had substantially overlapping confidence intervals (Tables S16 and S22, Supplementary Materials). Among the four original MHC variants, the largest within-window pairwise r^2^ was 0.019, and five of the six comparisons exceeded the clumping threshold of 0.001. The original selection therefore did not fully meet that threshold in East Asian populations, although correlation-aware models produced similar estimates (Tables S7 and S8, Supplementary Materials). Repeating the whole initial screen on the same basis gave the same conclusion. When all 12 datasets were re-selected against the East Asian panel and the false-discovery-rate correction was reapplied across the 12 all-variant tests, ulcerative colitis was again the only exposure that remained significant (q = 1.37 × 10^−4^, compared with q = 5.27 × 10^−4^ in the database-derived screen), and the next smallest corrected value was 0.347. No exposure changed its significance verdict (Table S5, Supplementary Materials).

## 4. Discussion

Ulcerative colitis was the only screened immune phenotype that remained associated with gastric cancer after correction when instruments were selected against an explicit East Asian linkage disequilibrium panel. The primary MHC estimate was similar to the all-variant estimate, whereas the primary non-MHC estimate was close to 1 but was based on only two variants and remained imprecise. The database-derived characterization instrument produced the same pattern. Ulcerative colitis and gastric cancer had overlapping association signals at *HLA-DQA1/DQB1*, and the MHC variants were also associated with several other immune diseases. Taken together, the association appears to be largely carried by pleiotropic MHC variants rather than to reflect a demonstrated causal effect of ulcerative colitis on gastric cancer, although the present data cannot establish which pathway explains it.

The MHC findings explain why the overall estimate should not be interpreted as an effect of ulcerative colitis as a clinical disease. HLA variants influence antigen presentation and susceptibility to many immune and infectious diseases, so variants selected for ulcerative colitis can be associated with gastric cancer through other pathways. In the primary analysis, the gastric cancer OR was 1.18 for the MHC group and 1.00 for the two-variant non-MHC group; the database-derived characterization estimates were 1.15 and 0.98, respectively. This pattern suggests that the positive estimate was most apparent in the MHC group, but it does not place ulcerative colitis on the causal pathway.

The cross-disease results support this cautious interpretation. The MHC variants were associated with several immune diseases and therefore showed limited specificity for ulcerative colitis. Non-MHC variants were also associated with systemic lupus erythematosus and asthma, showing that cross-disease associations were not restricted to the MHC. Associations with inflammatory bowel disease and Crohn’s disease could not provide independent confirmation because participants overlapped with the ulcerative colitis dataset. These cross-disease results should be interpreted within their limits. They provide evidence that the selected variants are broadly pleiotropic across immune diseases and therefore support limited specificity for ulcerative colitis, but they do not identify the biological pathway or the specific shared mechanism responsible for the gastric cancer association.

The non-MHC estimate was inconclusive rather than negative. The primary non-MHC estimate was imprecise (OR 0.999, 95% CI 0.856–1.167; two variants), and the characterization analysis was similarly imprecise (OR 0.984, 95% CI 0.857–1.130; four variants). Both confidence intervals included modest reductions and increases in gastric cancer odds. Because the exposure study used a targeted immune array, these variants do not represent the full spectrum of non-MHC ulcerative colitis susceptibility. The data therefore do not exclude a smaller non-MHC effect. Neither analysis, however, establishes that ulcerative colitis itself causes gastric cancer.

The HLA-DQ analysis showed regional overlap but did not establish causality. The same region could influence ulcerative colitis and gastric cancer through ulcerative colitis, another immune mechanism, or separate pathways. The MHC contains multiple association signals and long-range correlations; denser East Asian data and conditional fine-mapping are needed before this association can be attributed to a specific allele or pathway.

Observational data do not show a corresponding increase in gastric cancer risk. A Korean population cohort reported lower, rather than higher, gastric cancer risk among patients with ulcerative colitis [7]. The study found that long-term 5-aminosalicylate use was associated with lower risk and suggested that a lower prevalence of *H. pylori* infection might explain this finding; however, *H. pylori* status was not measured [7]. These findings do not establish that the gastric cancer association is mediated by ulcerative colitis. Alternative immune pathways remain possible, but the present analyses cannot distinguish among these explanations. *H. pylori* is a major environmental cause of gastric cancer in East Asia, and both host HLA susceptibility and antigen-driven immune modulation have been described [13,24]. The shared MHC signal could therefore reflect host responses to infection, but this remains an untested hypothesis because no *H. pylori*-related phenotype was analyzed here. Ancestry-matched exposure data would be required to distinguish this possibility from other shared MHC pathways.

The odds ratios describe changes in gastric cancer odds per unit increase in genetic liability to ulcerative colitis; they do not estimate the observed cancer risk of patients with ulcerative colitis. The positive estimate was observed with variants in the HLA region that were also associated with type 1 diabetes, Graves’ disease, rheumatoid arthritis, and multiple sclerosis. These cross-disease associations indicate limited specificity for ulcerative colitis but do not determine whether the gastric cancer association is mediated by ulcerative colitis or by another pathway. This genetic association does not support using ulcerative colitis alone as an indication for additional gastric cancer surveillance. Screening and surveillance should instead follow regional programs and established gastric cancer risk factors, including *H. pylori* infection, gastric atrophy or intestinal metaplasia, family history, and age [25]. When a genetic association between an immune disease and cancer is most apparent in HLA variants, shared susceptibility should be examined before the association is attributed to the disease itself.

A statistically significant IVW estimate can be misleading when the contributing variants lie in the MHC. A European-ancestry Mendelian randomization study reported no clear causal association and proposed shared genetic architecture as an alternative explanation [8]. In contrast, the previous East Asian analysis reported a positive ulcerative colitis–gastric cancer estimate but did not determine whether the estimate was specific to ulcerative colitis or most apparent in MHC variants [9]. In the present study, partitioning variants by MHC status and examining cross-disease associations provide a different interpretation of that positive estimate. The full screening results are reported because ulcerative colitis was selected after the initial screen rather than prespecified.

Several features strengthen the interpretation of our findings. The primary exposure and outcome summary statistics and the linkage disequilibrium reference used for instrument selection were all East Asian and came from different consortia, reducing ancestry mismatch in the association samples and making participant overlap unlikely. The complete initial screen is reported, including the post-screen selection of ulcerative colitis. Separating the MHC and non-MHC variants allowed us to examine whether the overall estimate was most apparent in the MHC and to directly show the uncertainty around the non-MHC estimate.

We checked the gastric cancer associations against the original GWAS Catalog file, quantified variant strength and residual MHC correlation, and obtained similar estimates with correlation-aware models. For the ulcerative colitis instrument, we used an explicit East Asian reference panel for primary selection and compared the results with the database-derived instrument. Across leave-one-out analyses, proxy exclusion, instrument re-selection, alternative estimators, regional overlap analyses, and cross-disease comparisons, the positive association remained most apparent in analyses that included MHC variants.

The main limitation is the size and coverage of the ulcerative colitis dataset. It included 1134 cases and used the Immunochip, leaving only two non-MHC variants in the primary East Asian-clumped instrument and four in the database-derived characterization instrument. The non-MHC confidence intervals therefore allowed for effects in either direction, and the targeted array may have missed relevant variants outside the MHC. This limitation is particularly important because the central MHC versus non-MHC comparison depends directly on these few non-MHC instruments. Variant selection and weighting used the same ulcerative colitis dataset, so winner’s curse may have inflated exposure estimates and F statistics. MR–Egger and the formal comparisons between MHC and non-MHC estimates were underpowered or unstable because so few variants were available. The MR-PRESSO global-test *p* value was near the 0.05 threshold with 100,000 simulations and therefore warrants cautious interpretation. With 80 percent power, the non-MHC analysis could detect effects corresponding to odds ratios of approximately 1.25 or 0.80 in the primary instrument and 1.22 or 0.82 in the characterization instrument, so a smaller effect in either direction cannot be excluded (Table S21, Supplementary Materials).

The reverse direction could not be examined: of ten gastric cancer variants selected against the East Asian panel, only one was present in the ulcerative colitis data and it was palindromic, leaving no interpretable estimate. The colocalization model assumed a single causal variant despite the likelihood of multiple MHC signals, and sparse ulcerative colitis coverage prevented a multi-signal analysis. The second gastric cancer dataset shared participants with the primary Biobank Japan dataset and therefore could not serve as an independent replication; we were unable to obtain suitable non-overlapping East Asian gastric cancer summary statistics for an independent replication in the present study, and subgroup analyses by anatomic site or histologic subtype were also unavailable. Given the biological heterogeneity of gastric cancer, independent East Asian replication would substantially strengthen these findings.

Most East Asian outcomes came from the same Biobank Japan release and were correlated. The inflammatory bowel disease and Crohn’s disease datasets shared cases or controls with the ulcerative colitis study and therefore did not provide independent confirmation. The European ulcerative colitis and multiple sclerosis datasets differed in ancestry from the primary analysis. The cross-disease associations indicate limited disease specificity but do not identify the pathway responsible for the gastric cancer association. Cross-trait analyses can help characterize shared genetic architecture across complex diseases [26]. Denser genome-wide ulcerative colitis data and East Asian linkage-disequilibrium information are needed to distinguish shared susceptibility from an effect mediated by ulcerative colitis. The non-MHC proxy was supported by European linkage disequilibrium but could not be confirmed in East Asian reference panels; excluding it produced similar estimates.

## 5. Conclusions

In East Asian populations, the association between genetic susceptibility to ulcerative colitis and gastric cancer persisted after explicit ancestry-matched instrument selection but was most apparent in MHC-containing instruments. The non-MHC estimate was close to 1 but was imprecise and did not exclude a moderate effect in either direction. The MHC variants were associated with several other immune diseases, and the overlapping signals at *HLA-DQA1/DQB1* did not identify a single causal variant or an ulcerative-colitis-mediated pathway. The association therefore appears to be largely carried by pleiotropic MHC variants; the present data do not identify the responsible pathway and do not show that ulcerative colitis itself increases gastric cancer risk. Independent replication in non-overlapping East Asian datasets with genome-wide exposure coverage is required.

## Figures and Tables

**Table 1:** Core data sources and focused analysis set.

Role	Trait	Dataset/Source	Ancestry	Sample Size	Analytical Use
Focused exposure	Ulcerative colitis	ieu-a-969; IIBDGC	East Asian	1134 cases/3719 controls	Primary: 6 EAS-clumped variants (4 MHC; 2 non-MHC). Expanded characterization: 8 database-derived variants (4; 4).
Primary outcome	Gastric cancer	ebi-a-GCST90018629; Biobank Japan	East Asian	7921 cases/159,201 controls	Source of all primary outcome estimates; different consortium from the exposure study.
Consistency outcome	Gastric cancer	bbj-a-119; Biobank Japan	East Asian	6563 cases/195,745 controls	Overlaps with the primary outcome and is not an independent replication.

Abbreviations: EAS, East Asian; IIBDGC, International Inflammatory Bowel Disease Genetics Consortium; MHC, major histocompatibility complex.

**Table 2:** Gastric cancer results by ulcerative colitis variant group.

Instrument and Variant Group	k	OR (95% CI)	*p*	Interpretation
East Asian-clumped primary: all variants	6	1.163 (1.087–1.244)	1.1 × 10^−5^	Significant after correction across 12 tests
East Asian-clumped primary: MHC variants	4	1.183 (1.111–1.259)	1.4 × 10^−7^	Similar to the all-variant estimate
East Asian-clumped primary: non-MHC variants	2	0.999 (0.856–1.167)	0.99	Imprecise; no clear association
Database-derived characterization: all variants	8	1.134 (1.068–1.205)	4.39 × 10^−5^	Same overall pattern
Database-derived characterization: MHC variants	4	1.154 (1.085–1.227)	4.92 × 10^−6^	Similar to the all-variant estimate
Database-derived characterization: non-MHC variants	4	0.984 (0.857–1.130)	0.823	Imprecise; no clear association

## Data Availability

Summary statistics were obtained from IEU OpenGWAS, https://gwas.mrcieu.ac.uk (accessed on 31 July 2026), and the GWAS Catalog, https://www.ebi.ac.uk/gwas/ (accessed on 31 July 2026). Accession numbers for all datasets are provided in Table S2, Supplementary Materials. No individual-level data were used. The analysis code, derived variant-level inputs, output tables, and random seeds are archived at Zenodo, https://zenodo.org/records/21736825 (accessed on 31 July 2026) under the digital object identifier 10.5281/zenodo.21736825, which is the version-specific identifier for version v1; the all-versions identifier for the same archive is 10.5281/zenodo.21736824. Supplementary tables and figures are provided as Supplementary Materials.

## Supplementary Materials

The following supporting information can be downloaded at: https://www.mdpi.com/article/10.3390/genes17091077/s1. Table S1: STROBE-MR checklist; Table S2: Data sources for the initial screen and focused analyses; Table S3: Database-derived instrument: complete immune-phenotype screen with all variants and after excluding MHC variants; Table S4: Database-derived instrument: complete initial screen using MHC variants only; Table S5: Complete primary screen using instruments selected against an East Asian reference panel; Table S6: Ulcerative colitis variants, strength, and analysis counts: database-derived characterization instrument; Table S7: Pairwise linkage disequilibrium among the analyzed ulcerative colitis variants of the database-derived characterization instrument in East Asians; Table S8: Gastric cancer results when MHC variants are included; Table S9: Statistical checks for the ulcerative colitis-gastric cancer results; Table S10: Overlap of association signals at *HLA-DQA1/DQB1*; Table S11: Sensitivity of the regional overlap analysis to prior probabilities; Table S12: Sensitivity of the regional overlap analysis to the analysis window; Table S13: Datasets used to examine associations with other diseases; Table S14: Sensitivity of selected estimates to alternative database retrieval; Table S15: Verification of outcome statistics against the original distributed summary data; Table S16: Gastric cancer results with the East Asian-clumped primary and database-derived characterization instruments; Table S17: Gastric cancer results and 95% confidence intervals; Table S18: Associations with intestinal, immune, and non-immune outcomes; Table S19: Leave-one-out results for the gastric cancer analysis; Table S20: Single-variant estimates for gastric cancer; Table S21: Observed precision and smallest detectable effect; Table S22: Instrument composition: database-derived characterization and East Asian-clumped primary instruments; Table S23: Variant-level details for the East Asian-clumped primary instrument; Figure S1: Complete initial immune-phenotype screen, database-derived instrument; Figure S2: Leave-one-out and single-variant results for gastric cancer.

## Abbreviations

The following abbreviations are used in this manuscript: CI, confidence interval; GWAS, genome-wide association study; IVW, inverse-variance weighted; LD, linkage disequilibrium; MHC, major histocompatibility complex; OR, odds ratio.
