# Supplementary material for: MHC-Related Shared Genetic Susceptibility Between Ulcerative Colitis and Gastric Cancer in East Asians
Source: Genes (Basel). 2026 Sep 7;17(9):1077. doi: 10.3390/genes17091077 (PMC13606914; doi:10.3390/genes17091077)
Supplement: Supplementary file 1 [file genes-17-01077-s001.zip › genes-4529025-supplementary.pdf]

Supplementary Materials: Tables and Figures

MHC-Related Shared Genetic Susceptibility Between Ulcerative Colitis and Gastric Cancer in East Asians

This supplement provides the complete East Asian linkage-disequilibrium-matched primary screen and the analyses supporting the ulcerative colitis results. It reports both the East Asian-clumped primary instrument and the database-derived characterization instrument, together with variant strength, linkage disequilibrium and proxy checks, regional analysis at HLA-DQA1/DQB1, statistical sensitivity analyses, cross-disease associations, and data-verification results.

Contents

Table S1. STROBE-MR checklist

Table S2. Data sources for the initial screen and focused analyses

Table S3. Database-derived instrument: complete immune-phenotype screen with all variants and after excluding MHC variants

Table S4. Database-derived instrument: complete initial screen using MHC variants only

Table S5. Complete primary screen using instruments selected against an East Asian reference panel

Table S6. Ulcerative colitis variants, strength, and analysis counts: database-derived characterization instrument

Table S7. Pairwise linkage disequilibrium among the analyzed ulcerative colitis variants of the database-derived characterization instrument in East Asians

Table S8. Gastric cancer results when MHC variants are included

Table S9. Statistical checks for the ulcerative colitis-gastric cancer results

Table S10. Overlap of association signals at HLA-DQA1/DQB1

Table S11. Sensitivity of the regional overlap analysis to prior probabilities

Table S12. Sensitivity of the regional overlap analysis to the analysis window

Table S13. Datasets used to examine associations with other diseases

Table S14. Sensitivity of selected estimates to alternative database retrieval

Table S15. Verification of outcome statistics against the original distributed summary data

Table S16. Gastric cancer results with the East Asian-clumped primary and database-derived characterization instruments

Table S17. Gastric cancer results and 95% confidence intervals

Table S18. Associations with intestinal, immune, and non-immune outcomes

Table S19. Leave-one-out results for the gastric cancer analysis

Table S20. Single-variant estimates for gastric cancer

Table S21. Observed precision and smallest detectable effect

Table S22. Instrument composition: database-derived characterization and East Asian-clumped primary instruments

Table S23. Variant-level details for the East Asian-clumped primary instrument

Figure S1. Complete initial immune-phenotype screen, database-derived instrument

Figure S2. Leave-one-out and single-variant results for gastric cancer

Table S1. STROBE-MR checklist

Item	Section	Checklist item	Location in manuscript
1	Title and abstract	Indicate Mendelian randomization (MR) as the study's design in the title and/or the abstract if that is a main purpose of the study.	Abstract, Methods: the two-stage summary-data design and use of MR estimators are stated.
2	Introduction – Background	Explain the scientific background and rationale. What is the exposure? Is a causal exposure–outcome relationship plausible? Justify why MR is helpful.	Introduction, paragraphs 1-3.
3	Introduction – Objectives	State specific objectives, including pre-specified causal hypotheses. State that MR estimates causal effects under specific assumptions.	Introduction, final paragraph: objective is to identify the source of the association without assuming causality.
4	Methods – Study design and data sources	Present key elements of the study design early; consider a table of data sources.	Methods, Study design and reporting; Data sources; Table 1 and Table S2.
4a	Setting	Study design and underlying population; setting, locations, dates.	Methods, Data sources and focused analysis set; Table S2. Data were accessed and verified in July 2026.
4b	Participants	Eligibility criteria, sources/methods of selection; sample size; any power/sample-size calculations.	Methods, Data sources and focused analysis set; Table 1. No a priori power calculation was performed; observed precision and the smallest detectable effect are reported in Table S21.
4c	Genetic variants	Describe measurement, quality control and selection of genetic variants.	Methods, Variant selection and MHC partitioning; Tables S6 and S14-S17.
4d	Variable assessment	Methods of assessment and diagnostic criteria for exposures, outcomes and other variables.	Methods, Data sources and focused analysis set; accessions and phenotype labels in Table S2.
4e	Ethics	Ethics committee approval and informed consent, if relevant.	Institutional Review Board Statement and Informed Consent Statement.

5	Methods – Assumptions	Explicitly state the three core IV assumptions (relevance, independence, exclusion restriction) and assumptions for additional/sensitivity analyses.	Methods, Variant selection and MHC partitioning: relevance, independence, and exclusion-restriction assumptions are stated explicitly.
6	Methods – Statistical methods (main)	Describe statistical methods and statistics used.	Methods, Association estimation and sensitivity analyses.
6a	Quantitative variables	How quantitative variables were handled (scale, units, model).	Methods, Association estimation and sensitivity analyses.
6b	Genetic variants / weights	How variants were handled and, if applicable, how weights were selected.	Methods, Variant selection and MHC partitioning.
6c	MR estimator	MR estimator (e.g., Wald ratio, IVW) and related statistics; covariates; two-sample covariate consistency.	Methods, Association estimation and sensitivity analyses.
6d	Missing data	How missing data were addressed.	Methods, Variant selection and MHC partitioning; missing variants are documented in Tables S6 and S15-S17.
6e	Multiple testing	If applicable, how multiple testing was addressed.	Methods, Association estimation and sensitivity analyses: Benjamini-Hochberg correction across the 12-test screen and 16 immune-disease comparisons.
7	Methods – Assessment of assumptions	Methods or prior knowledge used to assess assumptions or justify validity.	Methods, Variant selection and MHC partitioning; Association estimation and sensitivity analyses; Regional overlap of association signals.
8	Methods – Sensitivity and additional analyses	Describe sensitivity/additional analyses (alternative estimators, replication, bias analyses, instrument validation, simulations).	Methods, Association estimation and sensitivity analyses; Regional overlap of association signals; Associations with intestinal and other immune diseases.
9a	Methods – Software	Name statistical software and packages, with versions/settings.	Methods, Software and reproducibility; software packages are named, and the archived repository provides the analysis code, fixed input files, and outputs (https://doi.org/10.5281/zenodo.21736825

			for version v1; the all-versions identifier is 10.5281/zenodo.21736824).
9b	Methods – Pre-registration	State whether the protocol was pre-registered (when and where).	Methods, Study design and reporting: the protocol was not preregistered, and post-screen selection of ulcerative colitis is disclosed.
10a	Results – Descriptive data	Numbers of variants/individuals at each stage and reasons for exclusion (consider a flow diagram).	Methods and Results; Table 1; Tables S6 and S14-S17.
10b	Summary statistics	Summary statistics for exposures, outcomes and other variables.	Table 1 and Table S2.
10c	Meta-analysis heterogeneity	If data sources are meta-analyses, provide heterogeneity assessments.	Not applicable to the primary trait-level inputs.
10d	Two-sample MR	Justify similarity of variant–exposure associations across samples; report participant overlap.	Methods, Data sources; Table 1; Discussion, limitations.
11a	Results – Main results	Variant–exposure and variant–outcome associations on an interpretable scale.	Tables S6 and S14-S17.
11b	MR estimates	MR estimates and uncertainty on an interpretable scale (e.g., OR per SD).	Results; Table 2; Figures 2-4.
11c	Absolute risk	If relevant, translate relative into absolute risk.	Not applicable.
11d	Plots	Consider plots to visualize results (forest, scatter).	Figs. 2-4 and Figures S1-S2.
12a	Results – Assessment of assumptions	Report assessment of the validity of the assumptions.	Results; Tables S9-S10 and S18.
12b	Additional statistics	Additional statistics (e.g., I^2, Q statistic, E-value).	Results; Table S9.
13a	Results – Sensitivity/additional analyses	Sensitivity analyses assessing robustness to assumption violations.	Results; Tables S9-S12, S14, and S17-S21.
13b	Other sensitivity	Results from other sensitivity or additional analyses.	Results; Tables S14-S18 and S22.

13c	Direction of causal relationship	Assessment of direction (e.g., bidirectional MR).	Steiger analysis is reported in Table S9. The reverse direction was attempted but could not be estimated because only one gastric cancer variant was present in the ulcerative colitis data and it was palindromic. Steiger testing alone cannot establish directionality between two binary diseases.
13d	Comparison with non-MR	When relevant, compare with non-MR estimates.	Discussion compares observational evidence and prior MR reports.
13e	Additional plots	Additional plots (e.g., leave-one-out).	Leave-one-out and single-variant results are reported in Tables S19-S20 and Figure S2. Variant-level inputs are reported in Table S6.
14	Discussion – Key results	Summarize key results with reference to objectives.	Discussion, opening paragraph.
15	Discussion – Limitations	Discuss limitations: validity of IV assumptions, other bias, imprecision; direction and magnitude of bias and efforts to address it.	Discussion, limitations paragraphs.
16a	Discussion – Interpretation (meaning)	Cautious overall interpretation in context of limitations and other studies.	Discussion, paragraphs 2-5.
16b	Mechanism	Discuss biological mechanisms and gene–environment equivalence; use causal language carefully.	Discussion, MHC and H. pylori interpretation; causal language is explicitly limited.
16c	Clinical relevance	Whether results have clinical or policy relevance and inform intervention effect sizes.	Discussion: ulcerative colitis status alone does not support additional gastric cancer surveillance.
17	Discussion – Generalizability	Generalizability across populations, exposure periods and levels.	Discussion, limitations: East Asian focus, Immunochip coverage, outcome overlap, and ancestry mismatch.
18	Other – Funding	Sources of funding and role of funders, including for underlying databases.	Funding.
19	Other – Data and data sharing	Provide or state how data can be accessed; provide or state availability of statistical code.	Data Availability Statement.

20	Other – Conflicts of interest	All authors declare potential conflicts of interest.	Conflicts of Interest.

The checklist is included because the study uses Mendelian randomization estimators. The manuscript reports these results as genetic associations and does not claim a causal effect.

Table S2. Data sources for the initial screen and focused analyses

**Trait**	**OpenGWAS ID**	**Ancestry**	**N**
Gastric cancer (primary)	ebi-a-GCST90018629	East Asian	7921 / 159201
Gastric cancer (sensitivity)	bbj-a-119	East Asian	6563 / 195745
Asthma	ebi-a-GCST90018575	East Asian	13015 cases
Rheumatoid arthritis (GCST90018690)	ebi-a-GCST90018690	East Asian	5348 cases
Atopic dermatitis	ebi-a-GCST90018564	East Asian	4296 cases
Systemic lupus erythematosus	ebi-a-GCST90011866	East Asian	4222 cases
Rheumatoid arthritis (bbj-a-72)	bbj-a-72	East Asian	3636 cases
Medication use, thyroid preparations	ebi-a-GCST90018770	East Asian	3103 cases
Graves' disease	ebi-a-GCST90018627	East Asian	2809 cases
Crohn's disease	ieu-a-11	East Asian	1690 cases
Type 1 diabetes	ebi-a-GCST90018705	East Asian	1219 cases
Ulcerative colitis	ieu-a-969	East Asian	1134 cases
Hyperthyroidism	ebi-a-GCST90018640	East Asian	994 cases
Hashimoto thyroiditis	ebi-a-GCST90018635	East Asian	537 cases

The initial screen included 12 datasets representing 11 immune-related phenotypes, and results for all datasets are reported here. Ulcerative colitis was then examined in detail. The East Asian ulcerative colitis cohort (ieu-a-969) was genotyped using the Immunochip, which targets approximately 186 established immune-related

regions, whereas the gastric cancer studies used genome-wide arrays. Regional coverage therefore differed substantially: 280 variants were available for ulcerative colitis and 13,093 for gastric cancer in the HLA-DQA1/DQB1 region.

Table S3. Database-derived instrument: complete immune-phenotype screen with all variants and after excluding MHC variants

Exposure	MHC variants	k	OR	95% CI	P
Asthma	Included	25	1.014	0.942-1.092	0.708
Asthma	Excluded	21	1.004	0.915-1.102	0.938
Rheumatoid arthritis (GCST90018690)	Included	14	1.045	0.964-1.132	0.29
Rheumatoid arthritis (GCST90018690)	Excluded	6	0.865	0.731-1.024	0.0931
Atopic dermatitis	Included	16	0.913	0.851-0.980	0.0117
Atopic dermatitis	Excluded	12	0.958	0.888-1.034	0.274
Systemic lupus erythematosus	Included	31	1.041	1.001-1.082	0.0419
Systemic lupus erythematosus	Excluded	26	1.007	0.975-1.040	0.666
Rheumatoid arthritis (bbj-a-72)	Included	15	1.013	0.950-1.079	0.698
Rheumatoid arthritis (bbj-a-72)	Excluded	8	0.877	0.791-0.971	0.0114
Medication use, thyroid preparations	Included	6	1.118	0.999-1.252	0.0526
Medication use, thyroid preparations	Excluded	1	NE	NE	NE
Graves' disease	Included	18	1.021	0.981-1.064	0.311
Graves' disease	Excluded	11	1.005	0.944-1.071	0.867
Crohn's disease	Included	14	1.027	0.992-1.064	0.133
Crohn's disease	Excluded	9	1.006	0.980-1.032	0.656
Type 1 diabetes	Included	2	0.949	0.780-1.154	0.598
Type 1 diabetes	Excluded	1	NE	NE	NE
Ulcerative colitis	Included	8	1.134	1.068-1.205	4.39×10^{-5}
Ulcerative colitis	Excluded	4	0.984	0.857-1.130	0.823
Hyperthyroidism	Included	6	1.055	0.964-1.155	0.246
Hyperthyroidism	Excluded	1	NE	NE	NE
Hashimoto thyroiditis	Included	2	1.007	0.990-1.025	0.427
Hashimoto thyroiditis	Excluded	0	NE	NE	NE

Ulcerative colitis was examined in detail because it was the only association that remained significant after false-discovery-rate correction when MHC variants were included. Results for all 12 datasets are shown.

Table S4. Database-derived instrument: complete initial screen using MHC variants only

Exposure	k (MHC variants)	OR	95% CI	P	Approximate P for difference*
Asthma	4	1.03	0.91-1.18	0.61	0.75
Rheumatoid arthritis (GCST90018690)	8	1.07	0.97-1.17	0.17	0.031
Atopic dermatitis	4	0.81	0.74-0.88	2.9×10^{-6}	0.0046
Systemic lupus erythematosus	5	1.12	1.00-1.26	0.056	0.083
Rheumatoid arthritis (bbj-a-72)	7	1.04	0.97-1.12	0.24	0.0076
Medication use, thyroid preparations	5	1.15	1.04-1.28	0.0072	NE
Graves' disease	7	1.03	0.97-1.09	0.36	0.58
Crohn's disease	5	1.06	0.98-1.15	0.14	0.22
Type 1 diabetes	1	NE	NE	NE	NE
Ulcerative colitis	4	1.15	1.09-1.23	4.9×10^{-6}	0.04
Hyperthyroidism	5	1.07	0.97-1.18	0.19	NE
Hashimoto thyroiditis	2	1.01	0.99-1.02	0.43	NE

*P values comparing the MHC and non-MHC estimates use an exploratory zero-covariance approximation, are unadjusted, and do not account for possible covariance between estimates. They are not the primary evidence for a difference; alternative comparison methods for ulcerative colitis produced P values from 0.003 to 0.15.

Table S5. Complete primary screen using instruments selected against an East Asian reference panel

Exposure	k, database-derived	OR, database-derived	q, database-derived	k, East Asian-clumped primary instrument	OR, East Asian-clumped primary instrument	q, East Asian-clumped primary instrument
Ulcerative colitis	8	1.134	5.27×10^{-4}	6	1.163	1.37×10^{-4}
Medication use, thyroid preparations	6	1.118	0.158	4	1.119	0.347
Crohn's disease	14	1.027	0.319	10	1.026	0.416
Atopic dermatitis	16	0.913	0.070	18	0.943	0.418
Systemic lupus erythematosus	31	1.041	0.158	29	1.022	0.477
Graves' disease	18	1.021	0.467	14	1.023	0.503
Hyperthyroidism	6	1.055	0.467	4	1.04	0.503
Rheumatoid arthritis (GCST90018690)	14	1.045	0.467	11	1.041	0.503
Asthma	25	1.014	0.708	22	1.034	0.639
Rheumatoid arthritis (bbj-a-72)	15	1.013	0.708	12	1.023	0.766
Type 1 diabetes	2	0.949	0.708	3	0.976	0.766
Hashimoto thyroiditis	2	1.007	0.569	2	0.998	0.931

All 12 datasets in the initial screen were re-selected against the 1000 Genomes East Asian reference panel using the same thresholds as the primary analysis ($P < 5\times10^{-8}$, $r^2 < 0.001$, 10,000 kb), with variants matched by GRCh37 coordinates. The re-selection retained 147 variants across the 12 datasets. Odds ratios are dispersion-constrained inverse-variance-weighted estimates that use all available variants for each exposure, and the Benjamini-Hochberg correction was applied across the 12 all-variant tests exactly as in the primary screen. Ulcerative colitis was the only exposure that remained significant after correction with either instrument ($q = 5.27\times10^{-4}$ with the discovery instrument and $q = 1.37\times10^{-4}$ after re-selection); the next smallest corrected value after re-selection was 0.347. No exposure changed its significance verdict.

Table S6. Ulcerative colitis variants, strength, and analysis counts: database-derived characterization instrument

SNP	Position	Region	EA/OA; EAF	Exposure beta (SE)	F	Analyzed	Note
rs2647015	6:32664093	MHC	G/T; 0.2054	0.621 (0.0524)	140.4	Yes	-
rs7741807	6:29680657	MHC	C/G; 0.1811	0.549 (0.0548)	100.2	Yes	-
rs2844623	6:31232543	MHC	T/C; 0.2176	0.509 (0.0523)	94.6	Yes	-
rs13203429	6:32744852	MHC	C/G; 0.5220	0.346 (0.0484)	51.2	No	palindromic with ambiguous allele frequency
rs2274200	6:33625696	MHC	C/T; 0.2522	0.293 (0.0520)	31.8	Yes	-
rs13397737	2:43506324	non-MHC	T/G; 0.0436	-1.106 (0.1758)	39.6	Yes	Proxy rs55770424; 23,098 bp; CEU r^2 = 0.966; EAS LD unavailable
rs2940716	10:50124724	non-MHC	A/G; 0.0001	3.118 (0.4996)	38.9	No	not available in gastric cancer outcome
rs79315643	8:10773414	non-MHC	G/A; 0.0947	-0.586 (0.0963)	37.0	Yes	-
rs10975003	9:5213687	non-MHC	C/T; 0.1889	0.323 (0.0557)	33.7	Yes	-
rs10465507	1:173162439	non-MHC	A/C; 0.0497	-0.799 (0.1397)	32.7	Yes	-

Ten ulcerative-colitis-associated variants were selected at $P < 5\times10^{-8}$ after clumping at $r^2 < 0.001$ within 10,000 kb; eight entered the database-derived characterization analysis of gastric cancer. rs2940716 was unavailable in

the gastric cancer dataset, and rs13203429 had ambiguous allele alignment. rs13397737 was represented by a proxy variant. That characterization analysis included four MHC and four non-MHC variants. The East Asian-clumped primary instrument is a different set of six variants (four MHC and two non-MHC) and is described in Table S22. For the database-derived characterization instrument, the minimum F statistics were 31.8, 31.8, and 32.7 for all, MHC, and non-MHC variants, respectively. For the East Asian-clumped primary instrument, the corresponding minima were 32.7, 50.2, and 32.7, and the variant-level values are given in Table S23. EA denotes effect allele; EAF, effect-allele frequency; OA, other allele; and SE, standard error. Because the exposure study used a targeted immune array, the table does not represent a complete genome-wide variant selection.

In the primary gastric cancer dataset, rs13397737 was represented by rs55770424. The variants are 23,098 bp apart and were strongly correlated in the 1000 Genomes European reference panel ($r^2 = 0.966$), but no pairwise estimate was returned for the queried East Asian panels. Table S17 therefore includes an analysis excluding this proxy.

Table S7. Pairwise linkage disequilibrium among the analyzed ulcerative colitis variants of the database-derived characterization instrument in East Asians

SNP	rs10465507	rs7741807	rs2844623	rs2647015	rs2274200	rs10975003
rs10465507	1.00000	0.00050	0.00022	0.00037	0.00006	0.01460
rs7741807	0.00050	1.00000	0.00858	0.00664	0.00343	0.00034
rs2844623	0.00022	0.00858	1.00000	0.01872	0.00637	0.00069
rs2647015	0.00037	0.00664	0.01872	1.00000	0.00053	0.00219
rs2274200	0.00006	0.00343	0.00637	0.00053	1.00000	0.00061
rs10975003	0.01460	0.00034	0.00069	0.00219	0.00061	1.00000

Pairwise linkage disequilibrium was calculated with the 1000 Genomes East Asian reference panel and is displayed as r^2. Six of the eight analyzed variants were available in the panel; rs13397737 and rs79315643 were not. Among the four MHC variants, all of which were on chromosome 6 and within the 10,000-kb clumping window, the largest pairwise r^2 was 0.01873 between rs2844623 and rs2647015, and five of the six comparisons exceeded the threshold of 0.001. Correlations between variants on different chromosomes were not used to assess compliance with the clumping threshold. Correlation-aware models used the signed correlations after alignment to the exposure effect alleles; accounting for these correlations did not materially change the estimates (Table S8).

Table S8. Gastric cancer results when MHC variants are included

Method	k	OR	95% CI	P
All 8 variants, IVW	8	1.134	1.068-1.205	4.39×10^{-5}
All 8 variants, accounting for MHC correlations	8	1.130	1.058-1.207	2.69×10^{-4}
4 MHC variants, IVW	4	1.154	1.085-1.227	4.92×10^{-6}
4 MHC variants, accounting for correlations	4	1.154	1.077-1.235	4.25×10^{-5}
4 MHC variants, MR-Egger with correlations	4	1.308	1.019-1.678	0.0349

The primary analyses assumed independence among the selected variants. Additional generalized IVW analyses used the signed correlation matrix for the four MHC variants. In the eight-variant analysis, this 4 × 4 MHC block was embedded in an otherwise identity matrix, so correlations involving non-MHC variants were assumed to be zero. The MHC odds ratio changed by 0.0003, and its confidence interval widened by 1.4%. The all-variant odds ratio changed from 1.134 to 1.130. The correlation-aware MR-Egger estimate is shown but is not interpreted because four variants are insufficient for a stable estimate.

Table S9. Statistical checks for the ulcerative colitis-gastric cancer results

Sensitivity check	All variants	Non-MHC variants only
k	8	4
IVW result, characterization instrument, OR (P)	1.13 (4.4×10^{-5})	0.98 (0.82)
Cochran Q P	0.014	0.24
MR-Egger intercept P	0.29	0.25
MR-PRESSO global P (100,000 simulations)	0.049	0.43
Steiger result consistent with UC to gastric cancer direction	Yes	Yes
I^2(GX)	0.86	0.89
Weighted-median estimate, OR (P)	1.15 (4.52×10^{-7})	1.02 (0.80)
MR-Egger slope, OR (P)	1.28 (0.065)	0.68 (0.25)
MR-PRESSO outlier identified	rs2647015 (P = 0.045)	None
MR-PRESSO distortion test P	1.4×10^{-4}	Not applicable
MR-PRESSO outlier-corrected OR (native P)	1.099 (0.029)	Not applicable

These checks apply to the database-derived characterization instrument. The weighted-median estimate was close to the all-variant IVW estimate for that instrument, whereas the MR-Egger slope was imprecise. MR-Egger and MR-PRESSO had limited power with these variant counts. MR-PRESSO was run with 100,000 simulations. For the all-variant analysis, the global test remained at the significance boundary, the outlier test identified rs2647015, and the distortion test indicated that removing it changed the estimate. MR-PRESSO reported an outlier-corrected OR of 1.099 with its native P value of 0.029. The leave-one-out estimate after omitting the same variant was numerically identical but is reported separately in Table S19 because the two procedures use different inferential

calculations. No outlier was identified in the non-MHC analysis. These diagnostic results are exploratory and are not interpreted as evidence of causality.

Table S10. Overlap of association signals at HLA-DQA1/DQB1

**Quantity**	**Value**
Trait pair	Ulcerative colitis vs gastric cancer
Shared variants	265
Prior p1	1×10^{-4}
Prior p2	1×10^{-4}
Prior p12	1×10^{-5}
PP.H0	1.07×10^{-29}
PP.H1	5.98×10^{-27}
PP.H2	2.06×10^{-5}
PP.H3	0.0105
PP.H4	0.9895

The coloc.abf analysis covered chromosome 6:32,450,000-32,800,000 (GRCh37). Under the single-causal-variant assumption, the posterior probability of a shared regional signal was 0.989. The ulcerative colitis and gastric cancer signals overlapped in this region, but the analysis cannot determine whether one allele or an ulcerative-colitis-mediated pathway explains both signals.

Table S11. Sensitivity of the regional overlap analysis to prior probabilities

The coloc.abf analysis used standard prior probabilities (p1 = p2 = 1×10^{-4} and p12 = 1×10^{-5}) and the standard case-control effect-size prior. All five posterior probabilities for the default analysis are reported in Table S10. In the sensitivity analysis below, p12 was varied while p1 and p2 were held at 1×10^{-4}, and p1 and p2 were varied jointly while p12 was held at 1×10^{-5}.

**p1**	**p2**	**p12**	**PP.H3**	PP.H4
1×10^{-4}	1×10^{-4}	1×10^{-6}	0.0961	0.9037
1×10^{-4}	1×10^{-4}	5×10^{-6}	0.0208	0.9791
1×10^{-4}	1×10^{-4}	1×10^{-5}	0.0105	0.9895
1×10^{-4}	1×10^{-4}	5×10^{-5}	0.0021	0.9979
1×10^{-4}	1×10^{-4}	1×10^{-4}	0.0011	0.9989
1×10^{-5}	1×10^{-5}	1×10^{-5}	0.000106	0.9999
5×10^{-5}	5×10^{-5}	1×10^{-5}	0.0027	0.9973
1×10^{-4}	1×10^{-4}	1×10^{-5}	0.0105	0.9895
5×10^{-4}	5×10^{-4}	1×10^{-5}	0.2100	0.7899
1×10^{-3}	1×10^{-3}	1×10^{-5}	0.5153	0.4846

Support for a shared regional signal remained strong when p12 ranged from 1×10^{-6} to 1×10^{-4} (posterior probability >0.90). The result was more sensitive to the single-trait priors: increasing p1 and p2 jointly to 1×10^{-3}, with p12 held at 1×10^{-5}, reduced the shared-signal probability to 0.48. The finding therefore depends on the selected priors and the single-causal-variant assumption.

Table S12. Sensitivity of the regional overlap analysis to the analysis window

**Analysis region**	**Span (kb)**	**Variants available in both studies**	**PP.H3**	**PP.H4**
Analysis region used in the manuscript	350	265	0.0105	0.9895
Lead variant ±100 kb	200	204	0.0105	0.9895
Lead variant ±250 kb	500	425	0.0137	0.9863
Lead variant ±500 kb	1000	1135	0.0688	0.9312
Lead variant ±1 Mb	2000	1494	0.0953	0.9047

The analysis was repeated in windows centered on the lead ulcerative colitis variant rs2647015 (chromosome 6:32,664,093, GRCh37), which was the lead variant in every window. Priors were held at p1 = p2 = 1×10^{-4} and p12 = 1×10^{-5}. The posterior probability of a shared signal ranged from 0.9047 to 0.9895 and declined as the window widened. This decline may reflect changes in variant coverage and the inclusion of additional association signals; the present analysis cannot determine the cause of this sensitivity. The results are therefore read as regional overlap and not as evidence for a single causal allele or for a pathway through ulcerative colitis.

Table S13. Datasets used to examine associations with other diseases

Comparison group	Outcome selected	**Accession**	**Trait label in source database**	**Ancestry**	**Sample size**
UC in European data	Ulcerative colitis (European dataset)	ieu-a-970	Ulcerative colitis	European	13,768 cases / 47,745 total
Related intestinal disease	Crohn's disease	ieu-a-11	Crohn's disease	East Asian	1,690 cases / 5,409 total
Related intestinal disease	Inflammatory bowel disease	ieu-a-293	Inflammatory bowel disease	East Asian	2,824 cases / 6,543 total
Other immune disease	Type 1 diabetes	ebi-a-GCST90018705	Type 1 diabetes	East Asian	1,219 cases / 133,251 total
Other immune disease	Graves' disease	ebi-a-GCST90018627	Graves' disease	East Asian	2,809 cases / 175,465 total
Other immune disease	Rheumatoid arthritis	ebi-a-GCST90018690	Rheumatoid arthritis	East Asian	5,348 cases / 178,616 total
Other immune disease	Hashimoto thyroiditis	ebi-a-GCST90018635	Hashimoto thyroiditis	East Asian	537 cases / 173,193 total
Other immune disease	Systemic lupus erythematosus	ebi-a-GCST90011866	Systemic lupus erythematosus	East Asian	4,222 cases / 12,653 total
Other immune disease	Multiple sclerosis	ieu-b-18	multiple sclerosis	European	47,429 cases / 115,803 total
Other immune disease	Asthma	ebi-a-GCST90018575	Asthma	East Asian	13,015 cases / 175,948 total
Other immune disease	Atopic dermatitis	ebi-a-GCST90018564	Atopic dermatitis	East Asian	4,296 cases / 168,103 total
Non-immune comparator	Height	ukb-e-50_EAS	Standing height	East Asian	N=2,697
Non-immune comparator	Body mass index	ebi-a-GCST90018727	Body mass index	East Asian	163,835 total

Datasets were selected from OpenGWAS, prioritizing East Asian datasets with the largest available case counts. The inflammatory bowel disease and Crohn's disease datasets shared cases or controls with the ulcerative colitis study and were treated as within-consortium checks rather than independent confirmation. The European ulcerative colitis dataset was included to examine transferability across ancestries.

Table S14. Sensitivity of selected estimates to alternative database retrieval

**Outcome**	Variant group	**k**	**OR (95% CI), coordinate-verified retrieval**	**OR (95% CI), identifier-based retrieval**	**Ratio of odds ratios**
Gastric cancer (primary)	All variants	8	1.134 (1.07-1.21)	1.104 (1.03-1.18)	1.028
Gastric cancer (primary)	MHC variants only	4	1.154 (1.09-1.23)	1.133 (1.06-1.21)	1.019
Gastric cancer (primary)	Non-MHC variants only	4	0.984 (0.86-1.13)	0.939 (0.84-1.05)	1.048
Gastric cancer (sensitivity)	All variants	8	1.123 (1.06-1.19)	1.092 (1.02-1.16)	1.028
Gastric cancer (sensitivity)	MHC variants only	4	1.144 (1.08-1.21)	1.121 (1.05-1.20)	1.020
Gastric cancer (sensitivity)	Non-MHC variants only	4	0.963 (0.85-1.09)	0.928 (0.83-1.04)	1.038
Type 1 diabetes	All variants	8	0.844 (0.75-0.95)	0.884 (0.77-1.02)	0.956
Type 1 diabetes	MHC variants only	4	0.806 (0.73-0.89)	0.842 (0.71-1.00)	0.957
Type 1 diabetes	Non-MHC variants only	4	1.212 (0.91-1.61)	1.172 (0.90-1.53)	1.035
Graves' disease	All variants	8	0.710 (0.58-0.87)	0.751 (0.60-0.94)	0.945

**Outcome**	Variant group	**k**	**OR (95% CI), coordinate-verified retrieval**	**OR (95% CI), identifier-based retrieval**	**Ratio of odds ratios**
Graves' disease	MHC variants only	4	0.673 (0.51-0.88)	0.713 (0.51-0.99)	0.944
Graves' disease	Non-MHC variants only	4	1.072 (0.88-1.30)	1.019 (0.85-1.22)	1.052
Rheumatoid arthritis	All variants	8	0.806 (0.71-0.92)	0.802 (0.69-0.93)	1.005
Rheumatoid arthritis	MHC variants only	4	0.790 (0.65-0.96)	0.788 (0.62-1.01)	1.002
Rheumatoid arthritis	Non-MHC variants only	4	0.954 (0.83-1.10)	0.892 (0.78-1.02)	1.069

Both columns use the same database and datasets but locate variants differently. Records verified by coordinate and allele within the source database were used in the manuscript. That check is separate from the comparison against the originally distributed summary file, which is reported in Table S15 and covers the variants listed there. The identifier-based function returned neighboring variants for rs2844623 and rs10975003; those results are shown only as a retrieval check. Table S15 reports verification against the original distributed file.

Table S15. Verification of outcome statistics against the original distributed summary data

SNP	Original distributed record (GRCh37)	Retrieved record	Position match	|Δbeta|	|ΔSE|	Verdict	Analyzed?
rs2647015	chr6:32664093 G/T eaf=0.1786 beta=+0.12095 se=0.02161	chr6:32664093 G/T eaf=0.1786 beta=+0.12095 se=0.02161	True	0.0	0.0	Identical	Yes
rs7741807	chr6:29680657 C/G eaf=0.1795 beta=+0.04580 se=0.02135	chr6:29680657 C/G eaf=0.1795 beta=+0.04580 se=0.02135	True	0.0	0.0	Identical	Yes
rs2844623	chr6:31232543 T/C eaf=0.2155 beta=+0.08685 se=0.02018	chr6:31232543 T/C eaf=0.2155 beta=+0.08685 se=0.02018	True	0.0	0.0	Identical	Yes
rs13203429	chr6:32744852 C/G eaf=0.5272 beta=+0.03959 se=0.01678	chr6:32744852 C/G eaf=0.5272 beta=+0.03959 se=0.01678	True	0.0	0.0	Identical	No
rs2274200	chr6:33625696 C/T eaf=0.2390 beta=+0.01508 se=0.01957	chr6:33625696 C/T eaf=0.2390 beta=+0.01508 se=0.01957	True	0.0	0.0	Identical	Yes
rs13397737	Not present	Not present	-	-	-	Not present	Yes (proxy)
rs2940716	Not present	Not present	-	-	-	Not present	No
rs79315643	chr8:10773414 G/A eaf=0.0061 beta=+0.00754 se=0.10820	chr8:10773414 G/A eaf=0.0061 beta=+0.00754 se=0.10820	True	0.0	0.0	Identical	Yes
rs10975003	chr9:5213687 C/T eaf=0.1856 beta=+0.00845 se=0.02166	chr9:5213687 C/T eaf=0.1856 beta=+0.00845 se=0.02166	True	0.0	0.0	Identical	Yes
rs10465507	chr1:173162439 A/C eaf=0.0028 beta=+0.18526 se=0.15665	chr1:173162439 A/C eaf=0.0028 beta=+0.18526 se=0.15665	True	0.0	0.0	Identical	Yes

Each of the ten variants selected for the database-derived characterization instrument was compared with the GRCh37 gastric cancer summary file distributed by the GWAS Catalog for GCST90018629. The final column states whether the variant entered the gastric cancer analysis. rs13203429 was retrieved and checked but was excluded during harmonisation because of ambiguous allele alignment; rs2940716 was not present in the outcome dataset; and rs13397737 entered the analysis through the proxy rs55770424. The proxy record used in place of rs13397737 (rs55770424) was supplied by the outcome database and was not independently verified against the distributed file; the analyses excluding this proxy that are reported in Table S17 address its influence on the estimates but do not replace that verification. The variants that are unique to the East Asian-clumped primary instrument (rs2076177, rs389883 and rs2114436) are not covered by this table; their harmonised outcome records are provided in the archived repository as R1_harmonised_new_instrument.csv. Chromosome, position, alleles, effect-allele frequency, beta, and standard error were identical at the reported precision for all eight variants present in the file. rs2940716 was absent and had already been excluded. rs13397737 was represented by rs55770424 and therefore does not appear under its own identifier. Verification included rs2844623 and rs10975003, for which an identifier-based database function returned neighboring variants.

Table S16. Gastric cancer results with the East Asian-clumped primary and database-derived characterization instruments

Variant group	Instrument	k	OR	95% CI	P
All variants	Database-derived characterization instrument	8	1.134	1.068-1.205	4.39×10^{-5}
All variants	East Asian-clumped primary instrument	6	1.163	1.087-1.244	1.14×10^{-5}
MHC variants only	Database-derived characterization instrument	4	1.154	1.085-1.227	4.92×10^{-6}
MHC variants only	East Asian-clumped primary instrument	4	1.183	1.111-1.259	1.37×10^{-7}
Non-MHC variants only	Database-derived characterization instrument	4	0.984	0.857-1.130	0.823
Non-MHC variants only	East Asian-clumped primary instrument	2	0.999	0.856-1.167	0.991

Both sets of estimates use dispersion-constrained inverse-variance-weighted regression. With the East Asian-clumped primary instrument, the weighted-median estimates were 1.144 (95% CI 1.078-1.213) for all variants and 1.161 (95% CI 1.099-1.227) for MHC variants, and the MR-Egger slopes were imprecise (1.232, 95% CI 0.883-1.718; and 1.172, 95% CI 0.799-1.720). The MR-Egger intercept gave no clear evidence of directional pleiotropy (P=0.75 for all variants). Point estimates differed by no more than 2.6 percent, their directions were consistent, and the corresponding confidence intervals overlapped substantially, although only three variants were shared between the two instruments.

Table S17. Gastric cancer results and 95% confidence intervals

Variant group	No. of variants	OR	95% CI	P	Interpretation
All variants	8	1.134	1.068-1.205	4.39×10^{-5}	Association observed in the initial screen
MHC variants only	4	1.154	1.085-1.227	4.92×10^{-6}	Higher gastric cancer odds; secondary analysis
Non-MHC variants only	4	0.984	0.857-1.130	0.823	Estimate close to 1.0; confidence interval allows lower or higher odds
All variants; proxy omitted	7	1.136	1.072-1.204	1.48×10^{-5}	Positive estimate remained after excluding the ancestry-uncertain proxy
Non-MHC variants only; proxy omitted	3	0.998	0.887-1.122	0.972	Similar result after excluding the proxy

Table S17 reports the observed odds ratios and 95% confidence intervals. The non-MHC estimates were close to 1.0, but their confidence intervals included modest decreases and increases in gastric cancer odds. Excluding the ancestry-uncertain proxy produced similar all-variant and non-MHC estimates.

Table S18. Associations with intestinal, immune, and non-immune outcomes

**Category**	**Outcome**	**Anc.**	Variant group	**k**	OR or beta*	**95% CI**	**P**	**BH q**
UC in European data	Ulcerative colitis (European dataset)	EUR	All variants	9	1.001	0.969-1.033	0.968	NA
UC in European data	Ulcerative colitis (European dataset)	EUR	MHC variants only	4	1.044	0.886-1.231	0.606	NA
UC in European data	Ulcerative colitis (European dataset)	EUR	Non-MHC variants only	5	0.999	0.964-1.035	0.949	NA
Related intestinal disease	Crohn's disease	EAS	All variants	9	1.042	0.714-1.519	0.833	NA
Related intestinal disease	Crohn's disease	EAS	MHC variants only	4	0.717	0.592-0.867	6.23×10^{-4}	NA
Related intestinal disease	Crohn's disease	EAS	Non-MHC variants only	5	1.870	1.272-2.748	0.001	NA
Related intestinal disease	Inflammatory bowel disease	EAS	All variants	9	1.557	1.270-1.909	2.03×10^{-5}	NA
Related intestinal disease	Inflammatory bowel disease	EAS	MHC variants only	4	1.292	1.185-1.409	6.45×10^{-9}	NA
Related intestinal disease	Inflammatory bowel disease	EAS	Non-MHC variants only	5	2.104	1.625-2.724	1.65×10^{-8}	NA
Other immune disease	Type 1 diabetes	EAS	All variants	8	0.844	0.752-0.948	0.004	NA

**Category**	**Outcome**	**Anc.**	Variant group	**k**	OR or beta*	**95% CI**	**P**	**BH q**
Other immune disease	Type 1 diabetes	EAS	MHC variants only	4	0.806	0.727-0.893	3.80×10^{-5}	0.000467
Other immune disease	Type 1 diabetes	EAS	Non-MHC variants only	4	1.212	0.911-1.614	0.187	0.3
Other immune disease	Graves' disease	EAS	All variants	8	0.710	0.581-0.868	8.24×10^{-4}	NA
Other immune disease	Graves' disease	EAS	MHC variants only	4	0.673	0.513-0.884	0.004	0.0172
Other immune disease	Graves' disease	EAS	Non-MHC variants only	4	1.072	0.885-1.299	0.477	0.581
Other immune disease	Rheumatoid arthritis	EAS	All variants	8	0.806	0.708-0.918	0.001	NA
Other immune disease	Rheumatoid arthritis	EAS	MHC variants only	4	0.790	0.648-0.961	0.019	0.05
Other immune disease	Rheumatoid arthritis	EAS	Non-MHC variants only	4	0.954	0.828-1.098	0.508	0.581
Other immune disease	Hashimoto thyroiditis	EAS	All variants	8	1.015	0.822-1.253	0.889	NA
Other immune disease	Hashimoto thyroiditis	EAS	MHC variants only	4	0.968	0.724-1.295	0.828	0.828
Other immune disease	Hashimoto thyroiditis	EAS	Non-MHC variants only	4	1.503	0.978-2.310	0.063	0.144

**Category**	**Outcome**	**Anc.**	Variant group	**k**	OR or beta*	**95% CI**	**P**	**BH q**
Other immune disease	Systemic lupus erythematosus	EAS	All variants	6	1.179	0.914-1.521	0.204	NA
Other immune disease	Systemic lupus erythematosus	EAS	MHC variants only	3	1.144	0.726-1.801	0.563	0.6
Other immune disease	Systemic lupus erythematosus	EAS	Non-MHC variants only	3	1.286	1.117-1.481	4.62×10^{-4}	0.00246
Other immune disease	Multiple sclerosis	EUR	All variants	8	0.998	0.941-1.057	0.938	NA
Other immune disease	Multiple sclerosis	EUR	MHC variants only	3	0.692	0.534-0.897	0.005	0.0172
Other immune disease	Multiple sclerosis	EUR	Non-MHC variants only	5	1.010	0.991-1.030	0.308	0.422
Other immune disease	Asthma	EAS	All variants	8	0.976	0.892-1.068	0.600	NA
Other immune disease	Asthma	EAS	MHC variants only	4	0.949	0.855-1.052	0.317	0.422
Other immune disease	Asthma	EAS	Non-MHC variants only	4	1.222	1.108-1.347	5.84×10^{-5}	0.000467
Other immune disease	Atopic dermatitis	EAS	All variants	8	1.113	1.027-1.205	0.009	NA
Other immune disease	Atopic dermatitis	EAS	MHC variants only	4	1.111	0.977-1.263	0.108	0.216

Category	Outcome	Anc.	Variant group	k	OR or beta*	95% CI	P	BH q
Other immune disease	Atopic dermatitis	EAS	Non-MHC variants only	4	1.128	0.951-1.339	0.166	0.295
Non-immune comparator	Height	EAS	All variants	9	0.0090	[-0.0229, 0.0409]	0.581	NA
Non-immune comparator	Height	EAS	MHC variants only	4	0.0142	[-0.0368, 0.0653]	0.585	NA
Non-immune comparator	Height	EAS	Non-MHC variants only	5	0.0056	[-0.0352, 0.0464]	0.787	NA
Non-immune comparator	Body mass index	EAS	All variants	8	0.0042	[-0.0132, 0.0216]	0.636	NA
Non-immune comparator	Body mass index	EAS	MHC variants only	4	0.0054	[-0.0196, 0.0304]	0.670	NA
Non-immune comparator	Body mass index	EAS	Non-MHC variants only	4	-0.0058	[-0.0412, 0.0296]	0.750	NA

The number of variants differs across outcomes because availability and harmonisation differ by dataset; k is the number of the ten database-derived variants that could be harmonised with each outcome. Binary outcomes are reported as odds ratios. Height and body mass index are continuous outcomes and are reported as beta coefficients in the units of the source datasets. The inflammatory bowel disease and Crohn's disease datasets include participants from the ulcerative colitis study and are within-consortium checks. The European ulcerative colitis dataset examines cross-ancestry transferability and is not an independent replication of the East Asian result. For the eight other immune diseases, Benjamini-Hochberg q values were calculated across the 16 MHC and non-MHC comparisons. Associations with other immune diseases show that the selected variants are not specific to ulcerative colitis; they do not identify an alternative pathway to gastric cancer.

Table S19. Leave-one-out results for the gastric cancer analysis

Variant group	Variant omitted	Region	k	OR	95% CI	P
All variants	rs2647015	MHC	7	1.099	1.030-1.173	0.004
All variants	rs7741807	MHC	7	1.151	1.071-1.236	1.32×10^{-4}
All variants	rs2844623	MHC	7	1.118	1.041-1.201	0.002
All variants	rs2274200	MHC	7	1.142	1.070-1.220	7×10^{-5}
All variants	rs13397737	non-MHC	7	1.136	1.072-1.204	1.48×10^{-5}
All variants	rs79315643	non-MHC	7	1.136	1.065-1.212	1.08×10^{-4}
All variants	rs10975003	non-MHC	7	1.145	1.075-1.220	2.87×10^{-5}
All variants	rs10465507	non-MHC	7	1.138	1.073-1.208	1.66×10^{-5}
All variants	None (all included)	-	8	1.134	1.068-1.205	4.39×10^{-5}
MHC variants only	rs2647015	MHC	3	1.122	1.050-1.200	7.21×10^{-4}
MHC variants only	rs7741807	MHC	3	1.181	1.107-1.260	4.07×10^{-7}
MHC variants only	rs2844623	MHC	3	1.142	1.050-1.242	0.002
MHC variants only	rs2274200	MHC	3	1.165	1.091-1.245	5.74×10^{-6}
MHC variants only	None (all included)	-	4	1.154	1.085-1.227	4.92×10^{-6}
Non-MHC variants only	rs13397737	non-MHC	3	0.998	0.887-1.122	0.972
Non-MHC variants only	rs79315643	non-MHC	3	0.984	0.823-1.177	0.860
Non-MHC variants only	rs10975003	non-MHC	3	0.844	0.641-1.112	0.229
Non-MHC variants only	rs10465507	non-MHC	3	1.006	0.869-1.165	0.936
Non-MHC variants only	None (all included)	-	4	0.984	0.857-1.130	0.823

Each row omits one variant and repeats the dispersion-constrained inverse-variance-weighted analysis. In the all-variant group the odds ratio ranged from 1.099 to 1.151 and every estimate remained significant. Omitting rs2647015 gave the lowest value. No leave-one-out estimate in the non-MHC group was significant.

Table S20. Single-variant estimates for gastric cancer

SNP	Region	EAF	Beta, ulcerative colitis	Beta, gastric cancer	OR	95% CI	P
rs2647015	MHC	0.2054	0.621166	0.120949	1.215	1.135-1.301	2.17×10^{-8}
rs7741807	MHC	0.1811	0.548562	0.0458018	1.087	1.007-1.173	0.032
rs2844623	MHC	0.2176	0.509173	0.0868505	1.186	1.097-1.282	1.68×10^{-5}
rs2274200	MHC	0.2522	0.293384	0.0150779	1.053	0.924-1.200	0.441
rs13397737	non-MHC	0.04359	-1.10646	0.782115	0.493	0.214-1.138	0.098
rs79315643	non-MHC	0.09473	-0.58572	0.00753913	0.987	0.687-1.418	0.944
rs10975003	non-MHC	0.1889	0.323021	0.00845474	1.027	0.900-1.171	0.696
rs10465507	non-MHC	0.04973	-0.799165	0.185258	0.793	0.540-1.165	0.237

Wald ratio estimates obtained by dividing the gastric cancer effect by the ulcerative colitis effect for each analyzed variant. Three MHC variants, rs2647015, rs2844623 and rs7741807, were nominally associated with gastric cancer at $P < 0.05$. No variant outside the MHC was. These single-variant P values are unadjusted for multiple testing.

Table S21. Observed precision and smallest detectable effect

Variant group	k	Standard error of the log odds ratio	Smallest detectable odds ratio, increase	Smallest detectable odds ratio, decrease
All variants, characterization instrument	8	0.0309	1.09	0.92
MHC variants only, characterization instrument	4	0.0313	1.09	0.92
Non-MHC variants only, characterization instrument	4	0.0706	1.22	0.82
All variants, East Asian-clumped primary instrument	6	0.0344	1.10	0.91
MHC variants only, East Asian-clumped primary instrument	4	0.0318	1.09	0.92
Non-MHC variants only, East Asian-clumped primary instrument	2	0.0790	1.25	0.80

The smallest detectable odds ratio is the value that the observed standard error would detect with 80 percent power at a two-sided alpha of 0.05. Values are reported separately for the database-derived characterization instrument and for the East Asian-clumped primary instrument; the smallest detectable odds ratio is not the same quantity as the range of effects excluded by the observed confidence interval. Instrument strength is not expressed as a proportion of variance explained. Variant selection and effect estimation used the same ulcerative colitis dataset, so winner's curse inflates that quantity, and the transformation to the liability scale requires a population prevalence that is not established for ulcerative colitis in East Asians.

Table S22. Instrument composition: database-derived characterization and East Asian-clumped primary instruments

SNP	Chromosome	Position (GRCh37)	Region	P for ulcerative colitis	Database-derived characterization instrument	East Asian-clumped primary instrument
rs10465507	1	173162439	non-MHC	9.87×10^{-9}	Yes	Yes
rs13397737	2	43506324	non-MHC	2.76×10^{-10}	Yes	No
rs7741807	6	29680657	MHC	7.57×10^{-24}	Yes	No
rs2076177	6	29693113	MHC	4.55×10^{-19}	No	Yes
rs2844623	6	31232543	MHC	1.35×10^{-22}	Yes	No
rs389883	6	31947460	MHC	4.35×10^{-18}	No	Yes
rs2114436	6	32235384	MHC	1.19×10^{-12}	No	Yes
rs2647015	6	32664093	MHC	7.09×10^{-33}	Yes	Yes
rs13203429	6	32744852	MHC	7.01×10^{-13}	Yes	No
rs2274200	6	33625696	MHC	1.60×10^{-8}	Yes	No
rs79315643	8	10773414	non-MHC	1.06×10^{-9}	Yes	No
rs10975003	9	5213687	non-MHC	6.08×10^{-9}	Yes	Yes
rs2940716	10	50124724	non-MHC	4.11×10^{-10}	Yes	No

All 561 variants associated with ulcerative colitis at $P < 5\times10^{-8}$ were retrieved without clumping and were then clumped at $r^2 < 0.001$ within 10,000 kb against the 1000 Genomes East Asian reference panel. Coordinates were matched on GRCh37 positions. The database-derived characterization column marks the ten variants selected before outcome-specific harmonisation; eight of them entered the gastric cancer analysis. Of the ten variants in the database-derived characterization instrument, seven were present in the reference panel; rs13397737, rs2940716, and rs79315643 were not, and all three have low allele frequencies. Re-selection retained six variants, three of which were also in the database-derived characterization instrument. Requesting East Asian clumping from the database interface returned the same variants as a European request, so that route was not used.

Table S23. Variant-level details for the East Asian-clumped primary instrument

SNP	Position (GRCh37)	Region	EA/OA	EAF	Exposure beta (SE)	F
rs10465507	1:173162439	non-MHC	A/C	0.0497	-0.799 (0.1397)	32.7
rs2076177	6:29693113	MHC	T/C	0.2357	0.455 (0.0512)	78.9
rs389883	6:31947460	MHC	T/G	0.6835	-0.417 (0.0483)	74.5
rs2114436	6:32235384	MHC	G/C	0.7361	-0.360 (0.0508)	50.2
rs2647015	6:32664093	MHC	G/T	0.2054	0.621 (0.0524)	140.4
rs10975003	9:5213687	non-MHC	C/T	0.1889	0.323 (0.0557)	33.7

The six variants of the East Asian-clumped primary instrument, with the exposure effect estimates used to construct the instrument. Variant strength is $F = (betaX/SEbetaX)^2$. Minimum F values are 32.7 for all variants, 50.2 for MHC variants, and 32.7 for variants outside the MHC. Values are taken from the harmonised instrument file R1_harmonised_new_instrument.csv in the archived repository. EA denotes effect allele; EAF, effect-allele frequency; OA, other allele; and SE, standard error.

Figure S1. Complete initial immune-phenotype screen, database-derived instrument

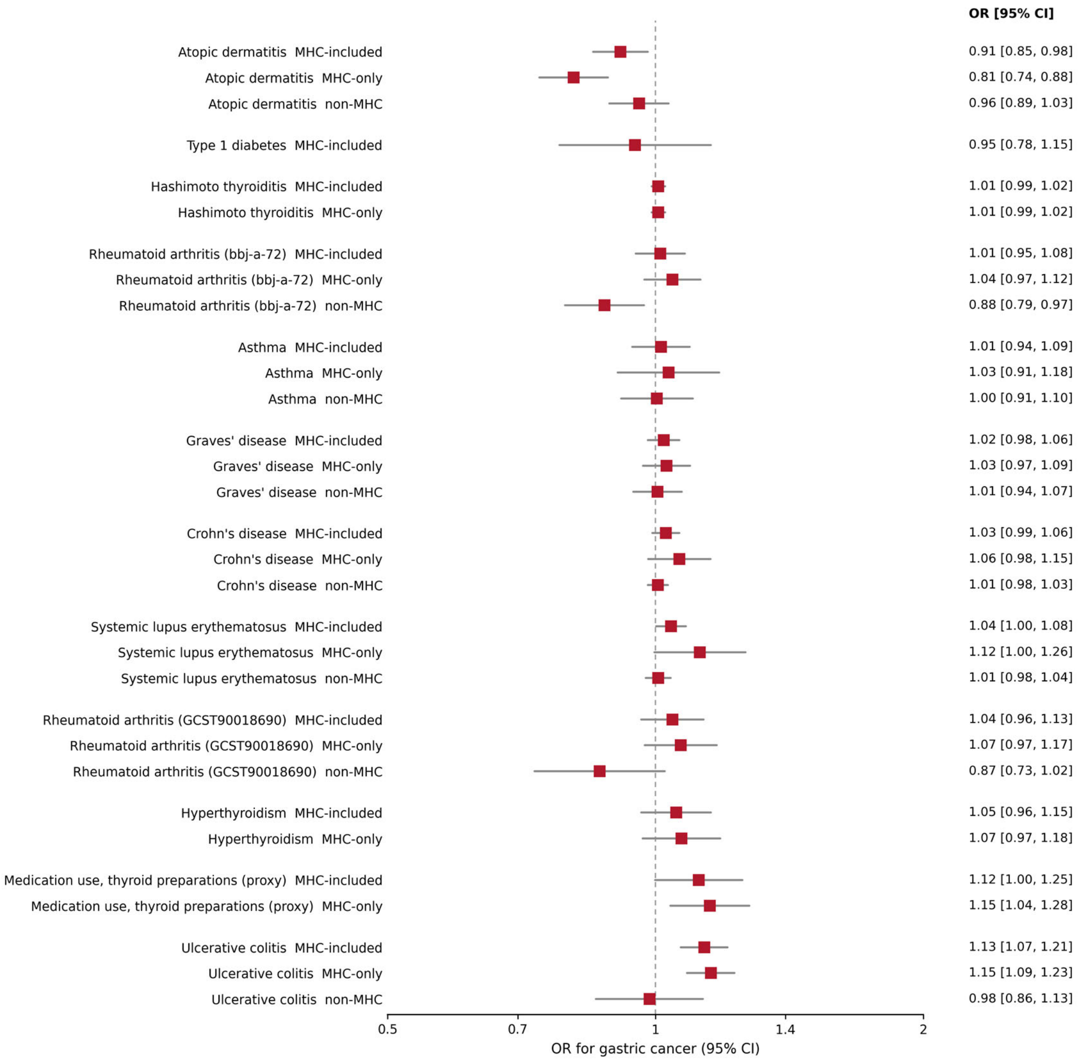

Figure S1. Results from the initial screen with the database-derived instrument, using all variants, MHC variants, and variants outside the MHC, where estimates could be calculated. The corresponding screen with instruments selected against the East Asian reference panel is reported in Table S5. Ulcerative colitis was the only phenotype selected for detailed evaluation after false-discovery-rate correction.

Figure S2. Leave-one-out and single-variant results for gastric cancer

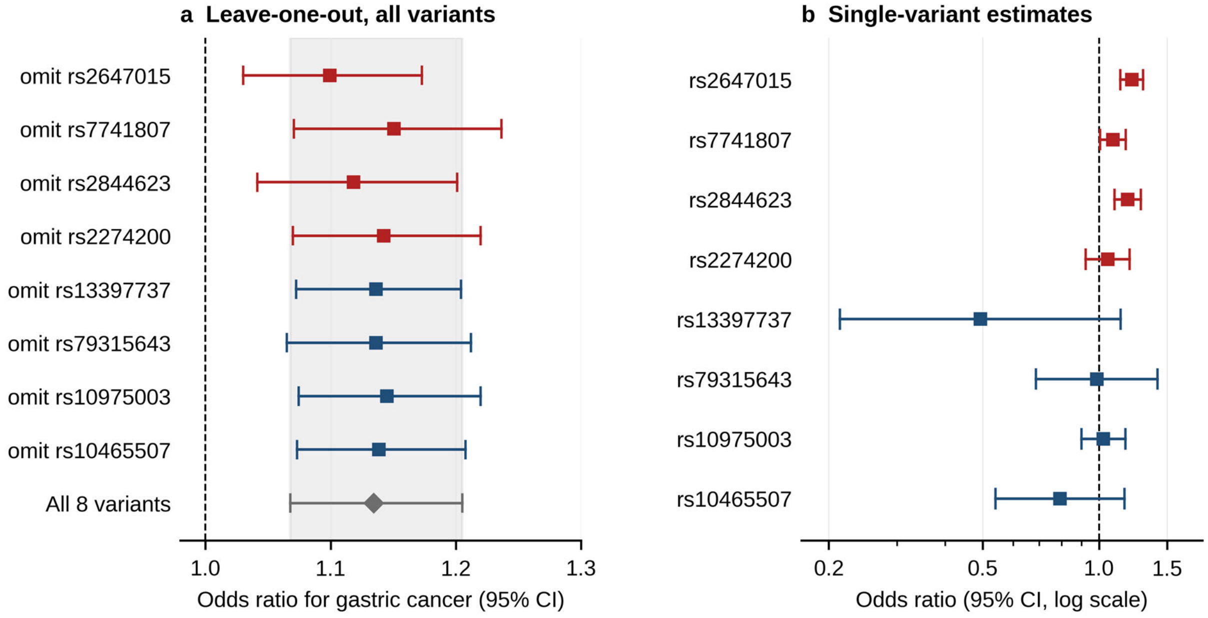

Figure S2. Leave-one-out and single-variant results for gastric cancer. Panel a omits one variant at a time from the all-variant analysis; the diamond and shaded band show the estimate and 95% confidence interval with all eight variants. Panel b shows Wald ratio estimates for each variant on a logarithmic scale. Red denotes MHC variants and blue denotes variants outside the MHC. Squares show odds ratios and horizontal lines 95% confidence intervals, and the dashed line marks an odds ratio of 1.0.
